# Circ_RPPH1 promotes bladder urothelium carcinoma proliferation and EMT by recruiting and binding to EIF4 A3

**DOI:** 10.1186/s41065-025-00442-3

**Published:** 2025-05-09

**Authors:** HuaWei Liu, JunMin Ma, Xia Yan

**Affiliations:** 1 Department of Urology, Deyang People’s Hospital, Sichuan Province, Deyang City, 618000 China; 2 Department of Urology, Huaian Hospital of Huaian City, Huaian City, Jiangsu Province 223200 China; 3 Department of Inspection Division, Xiangyang Central Hospital, Affiliated Hospital of Hubei University of Arts and Science, Hubei Province, No.1 Zhongyuan Road, Xiangyang City, 441003 China

**Keywords:** Circ_RPPH1, EIF4 A3, Bladder cancer, EMT

## Abstract

**Background:**

The involvement of circ_RPPH1 in bladder urothelial carcinoma (BUC) remains unclear, as well as the underlying mechanism.

**Methods:**

Circ_RPPH1 levels in BUC cells and tissues were measured via RT-qPCR. Downregulation of circ_RPPH1 was assessed using colony formation, CCK-8, wound healing, and Transwell assays to evaluate proliferation, migration, and invasion. RIP and RNA pull-down confirmed circ_RPPH1 binding to EIF4A3, while immunoblotting analyzed EIF4A3 and EMT-related proteins.

**Results:**

High circ_RPPH1 levels in BUC correlated with tumor invasion depth. Its knockout suppressed proliferation, invasion, and EMT, while circ_RPPH1 overexpression reduced EIF4A3 binding to N-cadherin and Vimentin mRNA, promoting EMT.

**Conclusion:**

Circ_RPPH1 promotes tumor growth and EMT in BUC by inhibiting EIF4A3-mediated mRNA regulation, activating the EIF4A3/N-cadherin/Vimentin pathway.

**Supplementary Information:**

The online version contains supplementary material available at 10.1186/s41065-025-00442-3.

## Introduction

Bladder cancer (BC) occurs in the urinary and reproductive systems. However, a prevalent subgroup of BC, bladder urothelial carcinoma (BUC) or bladder transitional cell carcinoma, accounts for over 90% of BC cases [1, 2]. The 5-year survival rate was 85% for patients with non-muscular invasive BC and 6% for those with muscular invasive BC because of distant metastases [3, 4]. Despite surgery, radiotherapy, and chemotherapy, invasive and metastatic BC tumors lead to high mortality [5]. With the change in living habits and population aging, the incidence of BUC increased year by year, with a high postoperative recurrence rate and poor prognosis. Therefore, it is a challenging task to further study BUC pathogenesis and find new therapeutic targets.

CircRNAs are endogenous non-coding RNAs that are ubiquitous across species [6]. The special structure of circRNAs can resist the hydrolysis of exonuclide and maintain high stability. CircRNAs were first identified in plant viruses in 1976 and are thought to be the product of a false splicing event [7]. CircRNAs can be protein-coding or non-coding [8, 9].Moreover, some circRNAs with certain structures are translatable, making them novel vaccines. Vaccines are efficient tools for immunotherapy [10]. However, circRNAs are now known to participate in cancers, including acting as miRNA sponges and ornamenting many transcription and translation processes [11]. A study reported that circ-Foxo3 induces apoptosis of BUC cells through interaction with miR-191-5p [12]. In addition, circ-FAM114 A2 inhibits BC progression by regulating NP63 through sponge miR-762 [13]. circ_RPPH1 is known to be involved in many cellular processes, including energy metabolism, proliferation, and tumorigenesis. For example, circRNA RPPH1 promotes BC progression by regulating miR-542-3p/ARHGAP1 pathway [14]. Also, circ_RPPH1 regulates glioma malignancy by inducing SDC1 expression by binding to miR-627-5p/miR-663a [15]. However, The mechanism of circ_RPPH1 in BUC has not been systematically explored. Our study reveals for the first time the mechanism by which circ_RPPH1 regulates BUC cell proliferation and EMT via EIF4 A3/N-cadherin/Vimentin pathway, filling the research gap in this field.

In summary, circ_RPPH1's high expression in BUC and its link to tumor progression highlight its significance, revealing a novel mechanism and offering a potential diagnostic and therapeutic target for BUC.

## Materials and methods

### Clinical tissue sample

In order to clarify the expression of circ_RPPH1 in clinical samples of bladder cancer, tumor tissues and paired adjacent normal tissues of 30 patients with bladder cancer were collected. All samples were obtained from the Institutional Review Committee of Deyang People's Hospital and confirmed by pathology. Patient selection criteria included clinical characteristics such as age, sex, tumor stage and grade, while excluding patients who had received preoperative chemotherapy or radiation therapy. Data collection included clinicopathological information and circ_RPPH1 expression level. Statistical analysis was performed using t test to evaluate the correlation between expression differences and clinical parameters. All patients signed informed consent forms,The study involving human samples was approved by the Institutional Review Committee of Deyang People’s Hospital (No.202103DY25).

### Cell culture

Human BUC cell lines T24, 5637, and J82 and normal urine epithelial cell line SV-HUC-1 were obtained from the ATCC. All cells were cultured in RPMI-1640 or DMEM containing 10% FBS (Gbico), 100 U/mL penicillin, and 100 μg/mL streptomycin (SigmaAldrich) and placed in a 5% carbon dioxide humidified incubator at 37 °C.

### RT-qPCR

MolPure® Cell/Tissue Total RNA Kit (YEASEN, Shanghai, China) was applied to extract total RNA. After assessing RNA concentration and purity using a NanoDrop 2000 spectrophotometer (Thermo Fisher Scientific, USA), the extracted RNA was conditioned to reverse transcription using M-MLV First Strand cDNA Synthesis kit (Beyotime, Beijing, China) and qPCR using SYBR Green qPCR SuperMix-UDG (Beyotime) in the 7300 real-time PCR system (Thermo Fisher Scientific). The amplification was performed under cyclic conditions with a total volume of 20 μL: 95 °C for 2 min, 95 °C for 15 s, 60 °C for 30 s, and 72 °C for 30 s. Circ_RPPH1 was normalized to GAPDH using the 2^−ΔΔCT^ method. Table 1 lists the primers.

**Table 1:** Primers

Genes	Forward	Reverse
Circ_RPPH1	5'-GGTCAGACTGGGCAGGAT-3'	5'-GAGTGACAGGACGCACTCAG-3'
GAPDH	5’-ATCTTCCAGGAGCGAGATCCC-3'	5'-TGAGTCTTCCACGATACCAA-3'

### Cell transfection

siRNAs of circ_RPPH1 (siRNA-circ_RPPH1-1: 5'-CAGGAGATGCCTGCGTCCTGT’3; siRNA-circ_RPPH1-2: 5'AGATGCCTGCGTCCTGTCACT-3') and EIF4 A3 (si-EIF4 A3: AGACAUGACUAAAGUGGAA) were obtained from Genpharma (Shanghai, China). Circ_RPPH1 sequences were inserted into pcDNA3.1 vector to produce an overexpression vector (Sangon, Shanghai, China). T24 and 5637 cells with 70% confluence were transfected using Lipo8000 reagent (Beyotime).

### Clonogenicity

Logarithmic T24 and 5637 cells were dispersed in 6-well plates at 500 cells/well and cultured with 2 mL medium containing 10% FBS (Gbico) for 14 days. Colonies that were visible were fixed in 4% paraformaldehyde, stained with crystal violet, photographed under a microscope (Olympus, Japan), and counted by ImageJ 1.52a.

### CCK-8 assay

T24 and 5637 cells were inoculated in 96-well plates at 2 × 10^3^ cells/well and placed under 5%CO_2_ at 37 °C. At 24, 48, 72, and 96 h post-transfection, 10 µL of CCK-8 reagent (Dojindo, Japan) was interacted with cells in each well for 2 h, thereby reading absorbance at 450 nm on a microplate reader (Bio-Rad, USA).

### Wound healing test

Three lines at the bottom of the 6-hole plate were drawn horizontally with 5 mm space between them. Three scratches were made on the T24 and 5637 cell layer perpendicular to the horizontal line using a 200μL pipette tip. Cells were photographed (0 h) and then photographed again after 36 h. The migration area was calculated using ImageJ 1.52a.

### Transwell invasion analysis

Transwell chambers (BD Biosciences, USA) were coated with matrix gel (BD Biosciences), and T24 and 5637 cells were collected 48 h after transfection to prepare cell suspension. The cells were resuspended in FBS-free DMEM, and 200 μl cell suspension (5 × 10^6^ cells/ml) was added to the upper compartment when 500 μl DMEM + 20% FBS was placed in the lower compartment. After 24 h, migratory and invasive cells were fixed with 4% paraformaldehyde and stained with 0.5% crystal violet, followed by cell counting under an inverted microscope (Olympus) in 5 visual fields.

### Immunoblottingting

T24 and 5637 cells were rinsed with pre-cooled PBS, and a lysis buffer (Beyotime, Shanghai, China) was added and placed on ice for 20 min to extract total proteins. After determining protein concentrations using the Lowry assay, the proteins were then isolated by 15% SDS-PAGE and transferred to PVDF membranes. At room temperature, 5% skim milk powder was incubated for 1 h, and the membrane was cleaned three times with TBST. The primary antibodies including Vimentin (92,547, rabbit monoclonal), E-cadherin (232,410, rabbit monoclonal), EIF4 A3 (32,485, rabbit polyclonal), N-cadherin (280,375, mouse monoclonal), and GAPDH (9485, Rabbit polyclonal) were incubated overnight at 4℃. After TBST washing, the secondary antibody (CST, USA) bound to the corresponding horseradish peroxidase was incubated at 37℃ for 1 h and developed using the ECL chemiluminescence kit (ultrassignal, China).

### RIP analysis

RIP testing was performed using a RIP kit (Millipore). The cell lysate products were detected with magnetic beads and anti-EIF4 A3 antibody or non-specific IgG. RNA was extracted from the complex and assessed by RT-qPCR.

### RNA pull-down test

T24 and 5637 cells were lysed to extract total protein by freezing and thawing in liquid nitrogen 3 times. Then, a circ_RPPH1 probe (Sangon) was added, as well as streptavidin magnetic beads, and incubated at 4 °C for 3 h. After the extraction of magnetic beads, EIF4 A3 was detected by immunoblotting.

### Data analysis

All data were analyzed using SPSS 25.0 and GraphPad Prism 8. The results were expressed as mean ± standard deviation. Two groups were compared by t test, and the clinical significance of circ_RPPH1 in BUC was evaluated by independent sample t test. *P* < 0.05 was considered statistically significant.

## Results

### Circ_RPPH1 expression is elevated in BUC tissues and is positively correlated with tumor invasion depth

**Fig. 1:**
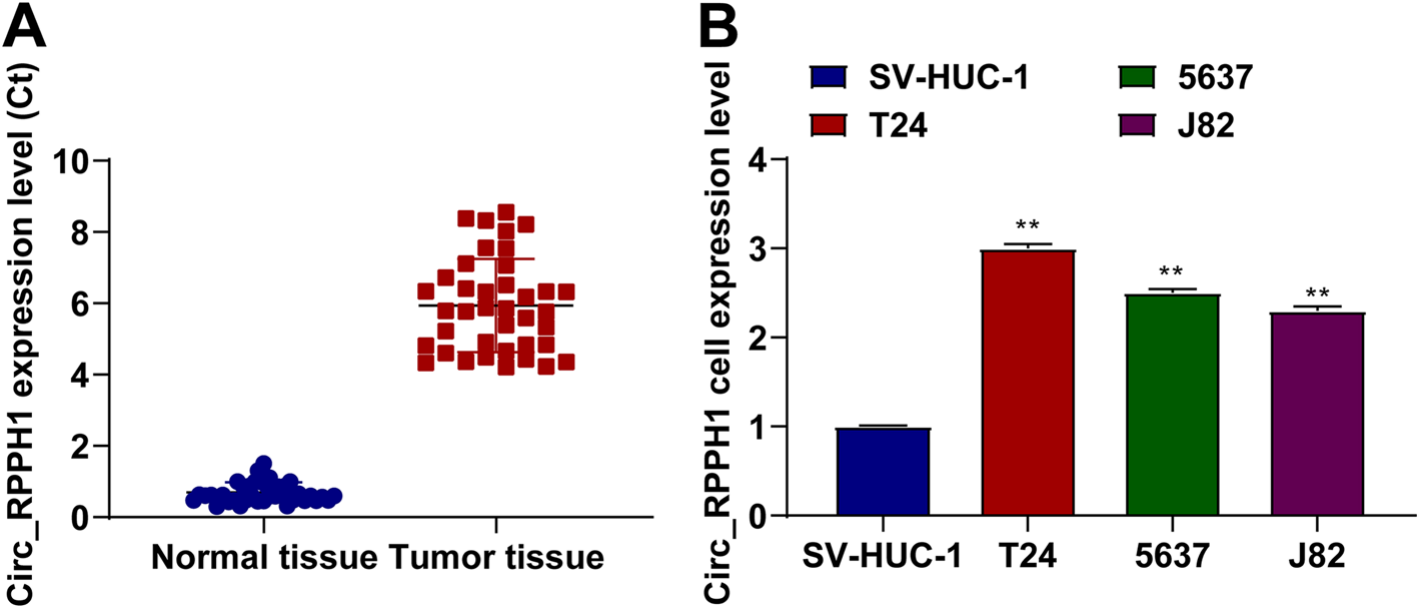
Circ_RPPH1 expression is elevated in BUC tissues and is positively correlated with tumor invasion depth. **A** Relative expression of circ_RPPH1 in BUC and adjacent normal bladder samples. **B** RT-qPCR detection of circ_RPPH1 mRNA levels in T24, 5637, J82, and SV-HUC-1 cell lines. Note: all experiment independently repeated three times, * *P* < 0.05, *P* < 0.01, *P* * * * * * < *P* < 0.0001, 0.001, * * * *

BUC tissues and normal tissues were harvested from 40 individuals, and circ_RPPH1 expression in BUC tissues was up-regulated (Fig. 1A). To verify this result, high expression of circ_RPPH1 was assessed in T24, 5637, J82, and SV-HUC-1 cells versus to SV-HUC-1 cells (Fig. 1B). Clinical data analysis presented that circ_RPPH1 suggested a high correlation with pathological TNM stage and grade, but not with age, tumor size, gender, and metastasis (Table 2).

**Table 2:** Relationship between expression of circ_RPPH1 and clinical factors in patients with BUC

**Clinical factors**	**Group**		Circ_RPPH1expression		
			High (%)	Low (%)	*p* Value
Gender	Male	35	31	4	0.094
	5	5	3	2	
Age	≤ 60	22	19	3	0.074
	> 60	18	15	3	
Tumor size	< 3 cm	10	9	1	0.261
	≥ 3 cm	30	25	5	
Invasive depth	pTa–PT1	7	5	2	0.02
	pT2–pT4	33	32	1	
Grade	High	33	30	3	0.002
	Low	7	3	4	
Metastasis	Yes	18	16	2	0.832
	No	22	20	2	

### Effective knockdown of circ_RPPH1 in T24 and 5637 cells

**Fig. 2:**
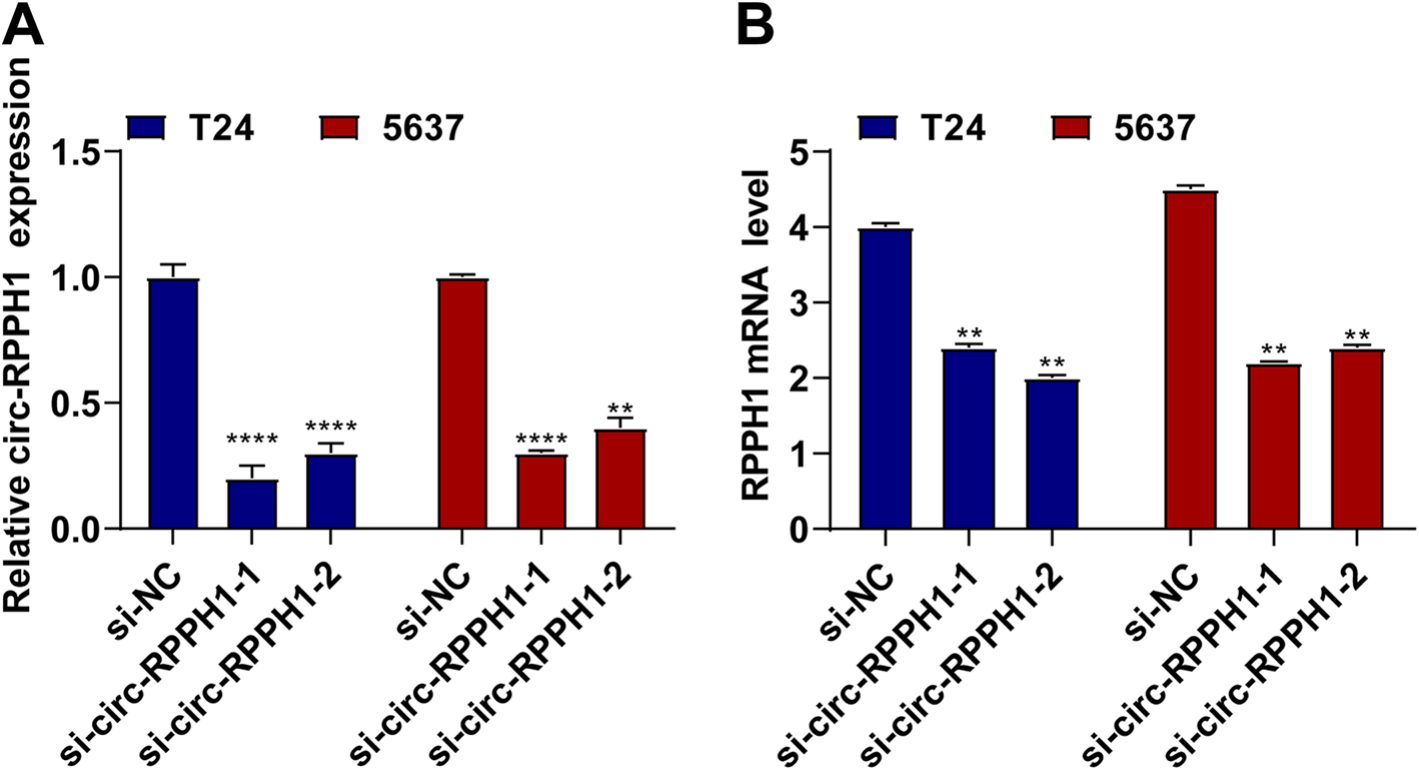
Fig. 2. Effective knockdown of circ_RPPH1 in T24 and 5637 cells **A **RT-qPCR detection of circ_RPPH1 in T24 and 5637 cells transfected with si-circ-RPPH1 **B** RT-qPCR detection of RPPH1 mRNA in T24 and 5637 cells transfected with si-circ-RPPH1.Note: all experiment independently repeated three times, * *P* < 0.05, *P *< 0.01, P * * * * * < *P *< 0.0001, 0.001, * * * *

si-circ_RPPH1 was transfected in T24 and 5637 cells to control circ_RPPH1 levels in BUC cells. The results proved that circ_RPPH1 was effectively silenced, with a greater efficiency achieved by siRNA-circ_RPPH1-1 compared with siRNA-circ_RPPH1-2 (Fig. 2A). Meanwhile, the study investigated no change in RPPH1 mRNA levels after circ-RPPH1 knockdown (Fig. 2B).

### Effects of circ-RPPH1 downregulation on the phenotype of BUC cells

**Fig. 3:**
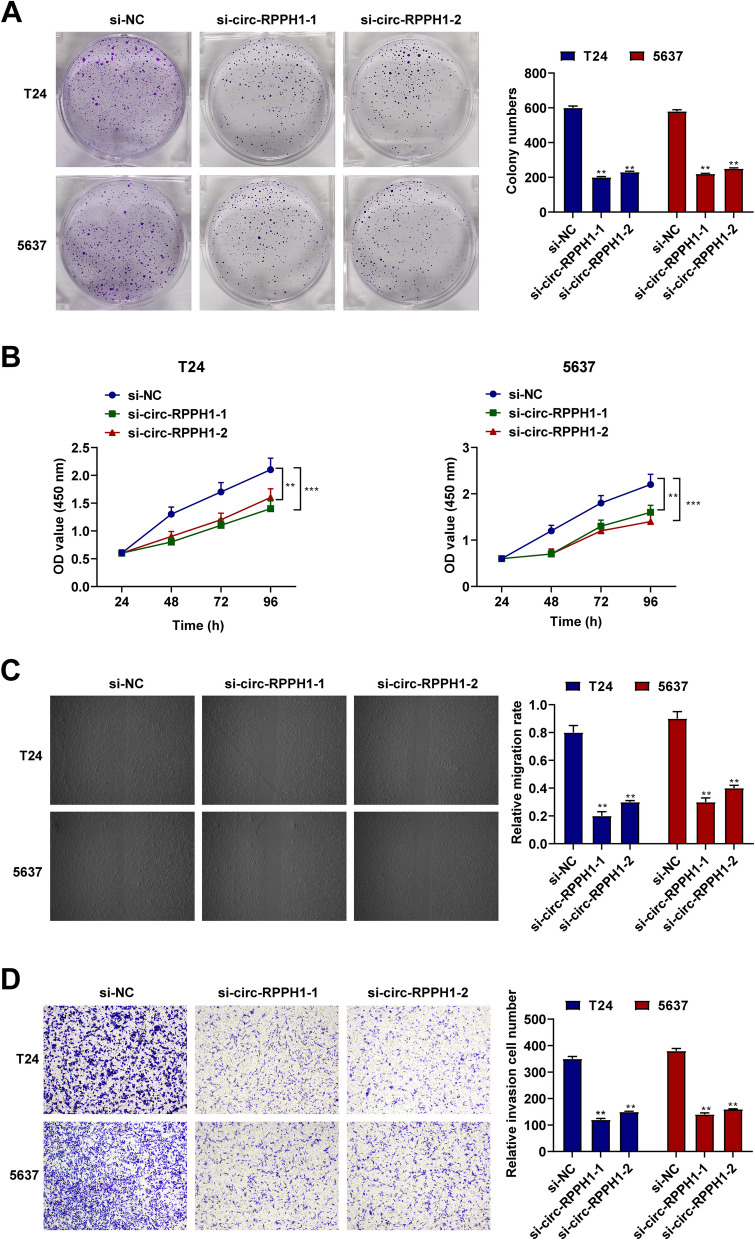
Effect of down-regulated circ-RPPH1 expression on BUC cell proliferation. **A** Colony formation analysis of the proliferation of T24 and 5637 cells. **B **CCK-8 assay detection of T24 and 5637 cell viability. **C** Transwell measured cell migration in T24 and 5637 cells. **D** Transwell measured invasion of T24 and 5637 cells. Note: all experiment independently repeated three times, * *P* < 0.05, *P *< 0.01, P * * * * * < *P *< 0.0001, 0.001, * * * *

Circ-RPPH1 was knocked out in BUC cells, and its effect on cell proliferation was checked by clonogenicity and CCK-8 assays. Reduction of circ-RPPH1 repressed the proliferative ability of T24 and 5637 cells (Fig. 3A, B). Secondly, the ability of BUC cells to migrate and invade was evaluated by wound healing and transwell tests. It was observed that knockout of circ-RPPH1 effectively inhibited cellular migration and invasion (Fig. 3C, D).

### Effect of down-regulated circ-RPPH1 on EMT of BUC cells

**Fig. 4:**
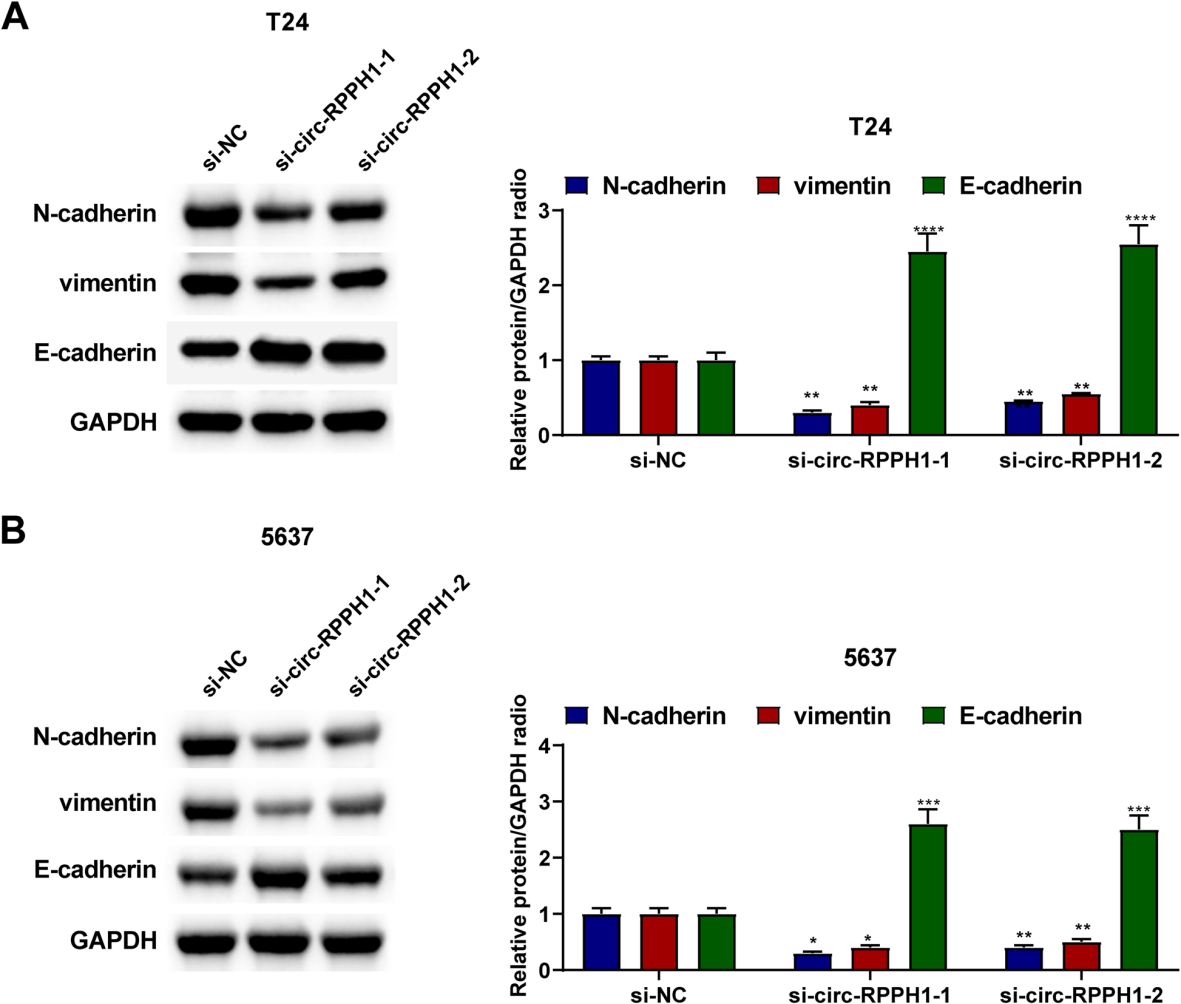
Effect of circ-RPPH1 down-regulation on EMT in BUC cells. Immunoblotting analysis of EMT proteins. Note: all experiment independently repeated three times, * *P *< 0.05, *P *< 0.01, P * * * * * < *P *< 0.0001, 0.001, * * * *

Since EMT confers the ability of stationary epithelial cells to migrate and invade, this study evaluated whether EMT-labeled proteins were altered following circ-RPPH1 knockdown. Immunoblottingting results demonstrated that after circ-RPPH1 knockout, N-cadherin and Vimentin protein expression was suppressed, while E-cadherin protein expression was enhanced (Fig. 4).

### Circ-RPPH1 may combine with EIF4 A3 to regulate EMT in BUC cells

**Fig. 5:**
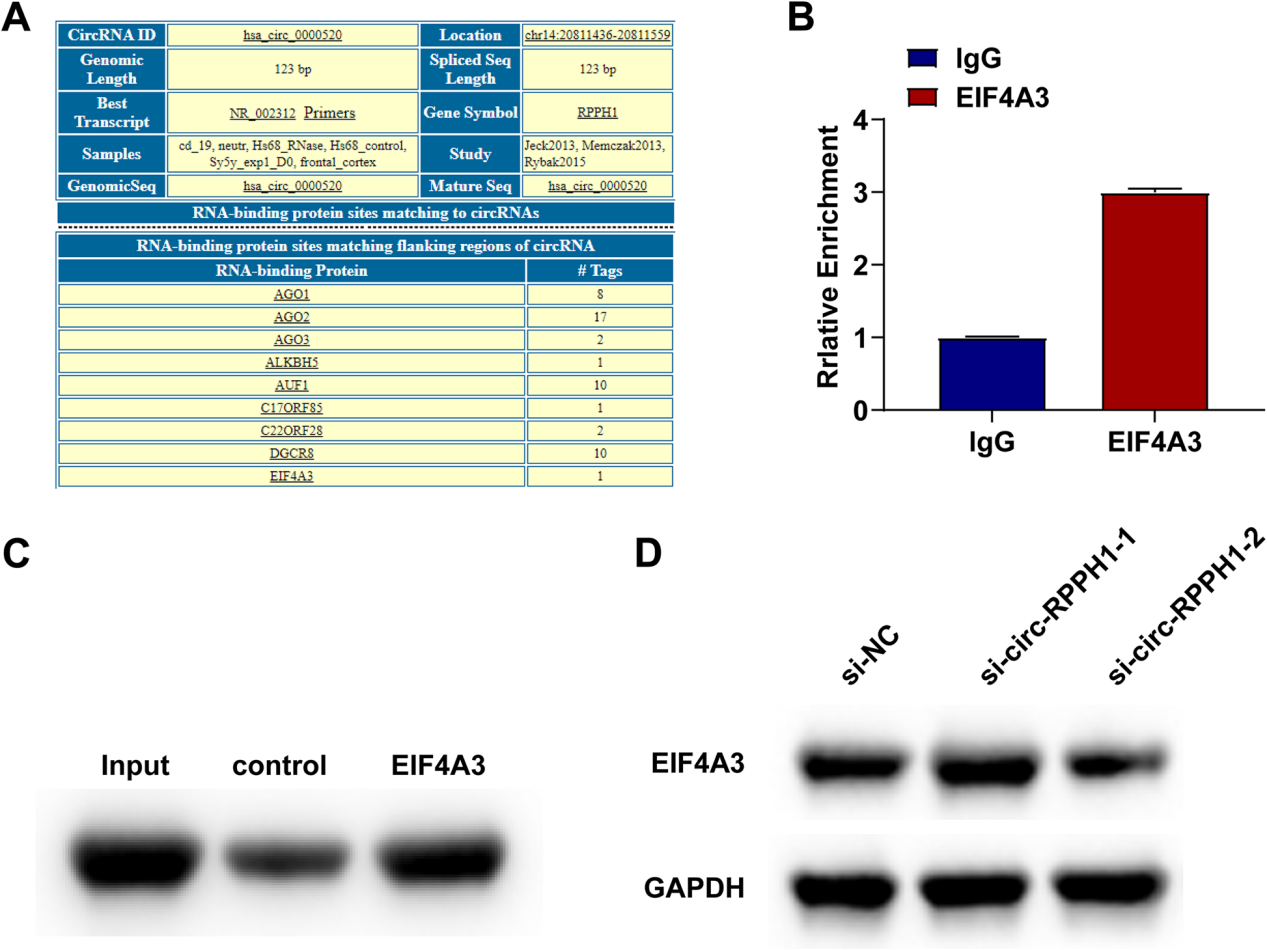
Circ-RPPH1 may combine with EIF4 A3 to regulate EMT in BUC cells. **A** Prediction of circ-RPPH1 binding to corresponding proteins by circInteractome. **B**-**C** RIP and RNA pull-down experiments showed that circ-RPPH1 binds to EIF4 A3. **D** Immunoblotting analysis of EIF4 A3 protein expression. Note: all experiment independently repeated three times, * *P *< 0.05, *P *< 0.01, P * * * * * < *P *< 0.0001, 0.001, * * * *

Non-coding RNAs can bind to specific proteins and affect tumor development [16, 17]. CircInteractome (https://circinteractome.nia.nih.gov) can predict proteins bound with circ-RPPH1, and only EIF4 A3 was selected for subsequent analysis (Fig. 5A). RIP experiment determined that circ-RPPH1 could interact with EIF4 A3 (Fig. 5B), and the binding of circ-RPPH1 with EIF4 A3 was further confirmed by RNA pull-down experiment (Fig. 5C). EIF4 A3 was detected after silencing circ-RPPH1 to investigate the existence of the regulatory correlation between EIF4 A3 and circ-RPPH1, finding no difference in EIF4 A3 expression (Fig. 5D).

### Circ-RPPH1 regulates EMT-related proteins by preventing EIF4 A3 from recruiting their respective mRNA

**Fig. 6:**
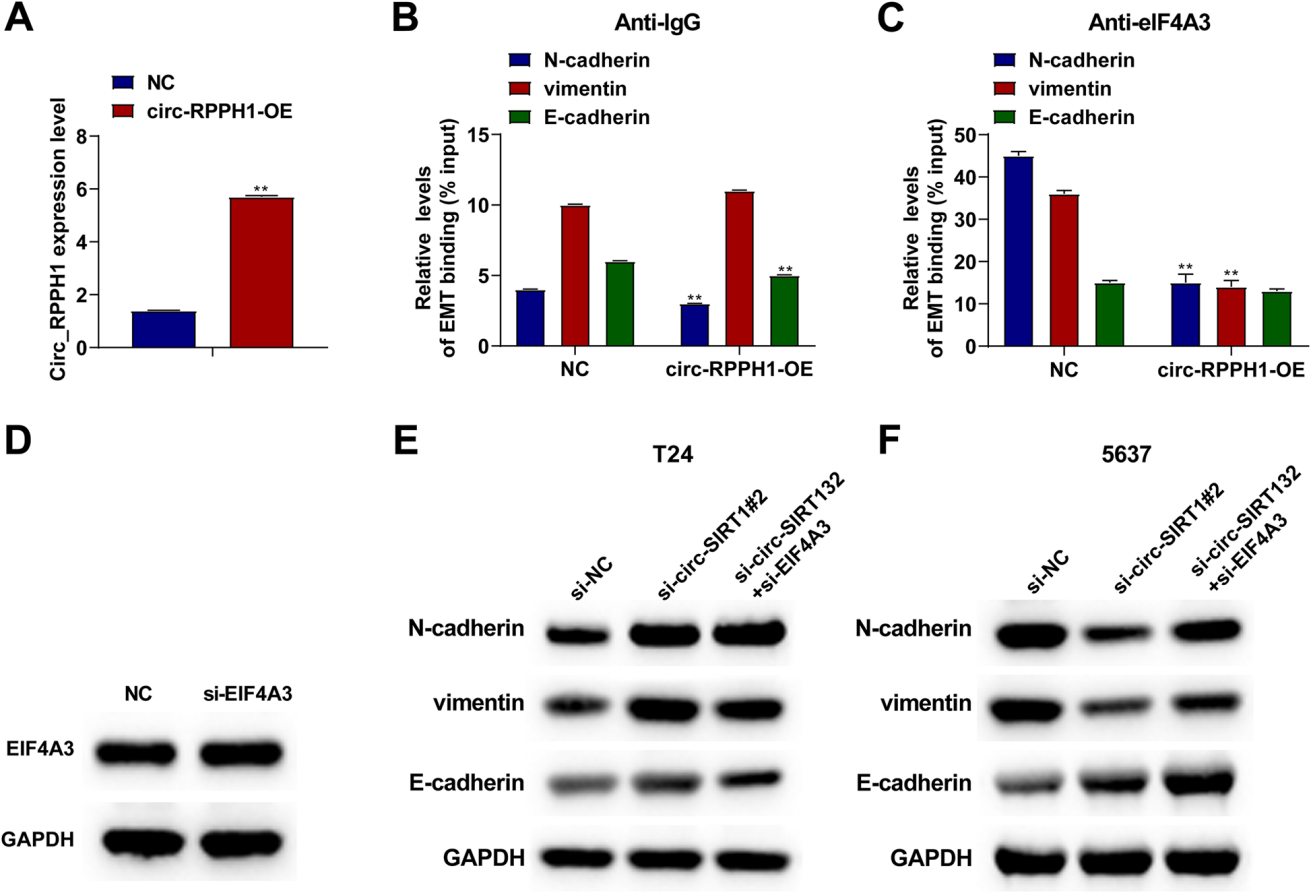
Circ-RPPH1 regulates EMT-related proteins by preventing EIF4 A3 from recruiting their respective mRNA. **A** RT-qPCR detection of circ-RPPH1 in T24 cells transfected with overexpressed vector. **B**-**C**. RIP detected EMT-related protein mRNA enrichment in EIF4 A3 RNA after circ-RPPH1 overexpression. **D**-**E** Immunoblotting analysis of EMT-related proteins. **F** Immunoblotting analysis of EMT protein after EIF4 A3 was down-regulated. Note: all experiment independently repeated three times, * *P *< 0.05, *P* < 0.01, *P* * * * * * < *P* < 0.0001, 0.001, * * * *

EIF4 A3 is involved in mRNA quality control prior to translation initiation [17]. Here, this study hypothesizes that circ-RPPH1/EIF4 A3 may influence EIF4 A3 abundance on EMT-labeled protein mRNA. After circ-RPPH1 overexpression (Fig. 6A), RIP assay was utilized to detect EIF4 A3 enrichment on EMT-related protein mRNA. Results showed less precipitation of N-cadherin and Vimentin mRNA in the anti-EIF4 A3 group (Fig. 6B, C), suggesting that circ-RPPH1 may regulate EMT-related marker proteins by preventing EIF4 A3 recruitment to the corresponding mRNA. To verify this, T24 cells were transfected with si-EIF4 A3 (Fig. 6D). Knockout of EIF4 A3 up-regulated N-cadherin and Vimentin but did not affect E-cadherin (Fig. 6E). Then, si-EIF4 A3 and si-circ-RPPH1-1 or si-circ-RPPH1-2 were co-transfected into T24 cells (Fig. 6F), and down-regulating EIF4 A3 reversed the down-regulating effect of circ-RPPH1 on EMT.

## Discussion

circRNA’s biological significance and function have received more and more attention, particularly in malignant tumors [18, 19]. BUC patients have been facing a great survival challenge due to tumor recurrence and metastasis. Early elucidation of the molecular mechanisms of BUC proliferation and metastasis may provide more treatment options for disease treatment.

RPPH1 expression has been tested to be up-regulated in BC [20]. Here, circ-RPPH1 expression levels in human BUC tissues were upregulated compared to paired and adjacent normal bladder samples. Postoperative pathological analysis of clinical patients demonstrated greater tumor invasion in patients with high circ-RPPH1 expression compared to patients with low circ-RPPH1 expression. However, this work did not observe a significant association between circ-RPPH1 and TNM staging and lymph node metastasis, possibly because of the small sample size. After circ-RPPH1 knockout, this study observed inhibition of proliferation, migration, and invasion in T24 and 5637 cells, suggesting that circ-RPPH1 serves as an oncogene in BUC.

EMT induces tumor cells to migrate and invade and constitutes the mechanistic interplay during tumor proliferation and metastasis [21–23]. The collected data demonstrated that N-cadherin and Vimentin were suppressed, while E-cadherin was elevated, suggesting that circ-RPPH1 absence could lead to the inhibition of EMT.

circRNAs can interact with proteins in tumors [24]. Here, this study used the CircInteractome to predict and select EIF4 A3 that could bind circ-RPPH1. In addition, circ-RPPH1 did not alter EIF4 A3 expression, suggesting that EIF4 A3 may be recruited by circ-RPPH1 to modulate downstream targets. EIF4 A3, a component of the exon junction complex (EJC), can influence protein translation and expression levels through a mechanism enabled by pre-mRNA splicing loading onto the mRNA and triggering sense-mediated mRNA decay [25, 26]. Therefore, it speculates that circ-RPPH1/EIF4 A3 may affect the abundance of EIF4 A3 on EMT-associated protein mRNA. Our idea was verified by RIP experiment results that In anti-EIF4 A3 antibody/RNA immunoprecipitation, N-cadherin and Vimentin mRNA precipitation levels decreased, while E-cadherin mRNA did not change significantly. This may be due to the different results in different mRNA regions of EJC. EIF4 A3 gene knockout results in significantly increased expression of activity-regulated cytoskeleton-associated proteins in somatic cells and dendrites [27]. To further examine the underlying mechanism, T24 cells were transfected with si-EIF4 A3 plus si-RNA-circ-RPPH1-1 and si-RNA-circ-RPPH1-2, and reported that si-EIF4 A3 can mitigate the inhibition effect of si-circ-RPPH1 on EMT.

## Conclusion

Circ-RPPH1 inhibits the recruitment of EMT-related protein mRNA by EIF4 A3, thus promoting the progression of EMT and proliferation of BUC cells. This study demonstrates for the first time that the circ-RPPH1/EIF4 A3/N-cadherin/Vimentin axis accelerates proliferation and EMT in BUC, providing new therapeutic targets and strategies for BUC treatment. However, the mechanism of circ-RPPH1/EIF4 A3/regulating E-cadherin expression needs further study.The shortcomings of this study, Sample Size and Clinical Correlations: The lack of association between circ-RPPH1 and TNM stage/lymph node metastasis may reflect insufficient statistical power. Future studies should expand patient cohorts and include longitudinal data to assess prognostic value. In addition, While we demonstrated circ-RPPH1's interaction with EIF4 A3, the precise molecular determinants of their binding specificity remain unclear. Structural studies could elucidate this.

## Supplementary Information

Supplementary Material 1.

## Data Availability

The datasets used and/or analyzed during the present study are available from the corresponding author on reasonable request.
