# Supplementary figures and images for: Circ_RPPH1 promotes bladder urothelium carcinoma proliferation and EMT by recruiting and binding to EIF4 A3
Source: Hereditas. 2025 May 9;162:72. doi: 10.1186/s41065-025-00442-3 (PMC12065329; doi:10.1186/s41065-025-00442-3)

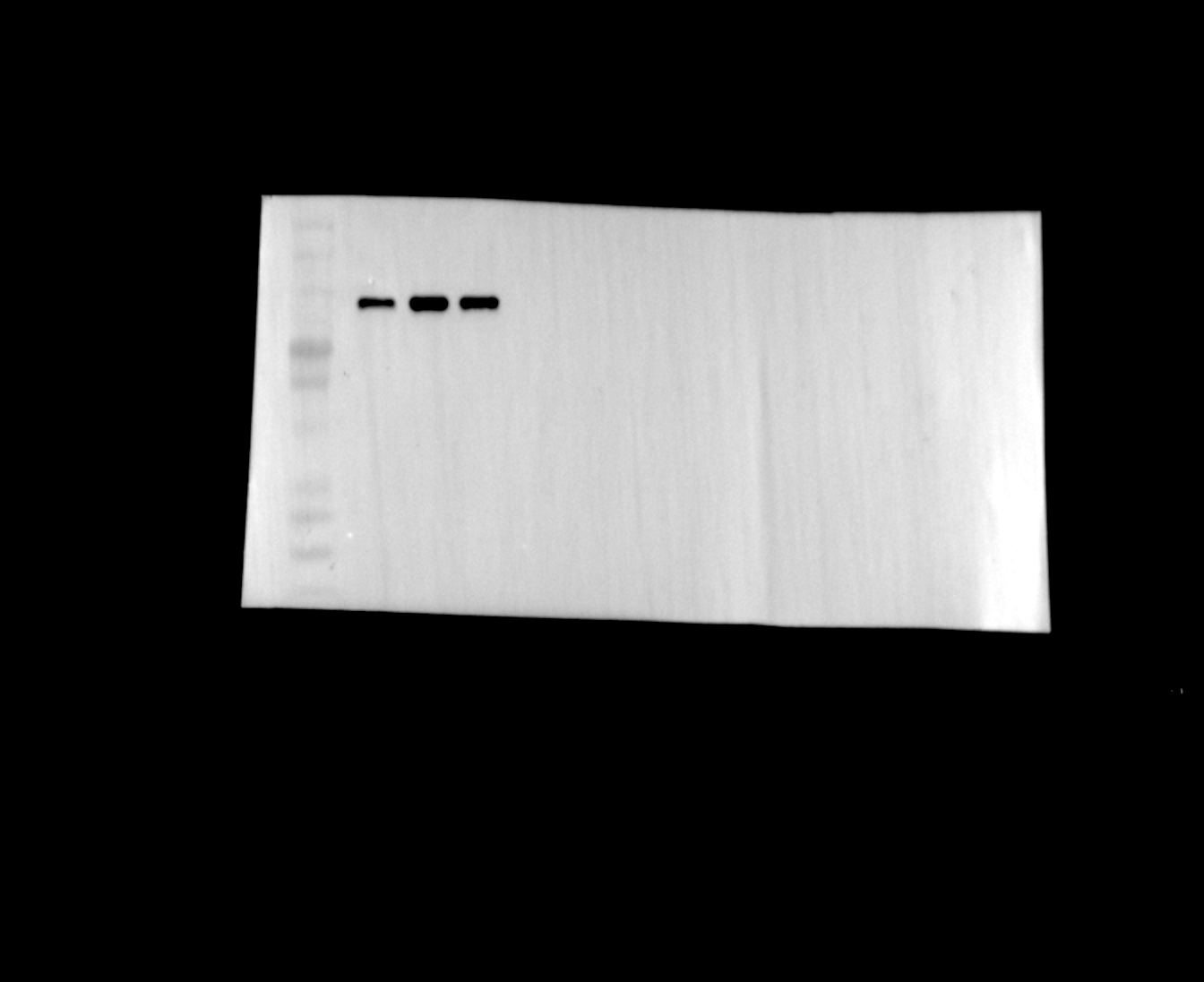

Supplement: Supplementary file 1 — Supplementary Material 1. [file 41065_2025_442_MOESM1_ESM.zip › Original image for western blot-marker/Original image Figure 4A/Figure 4A E-cadherin.tif]

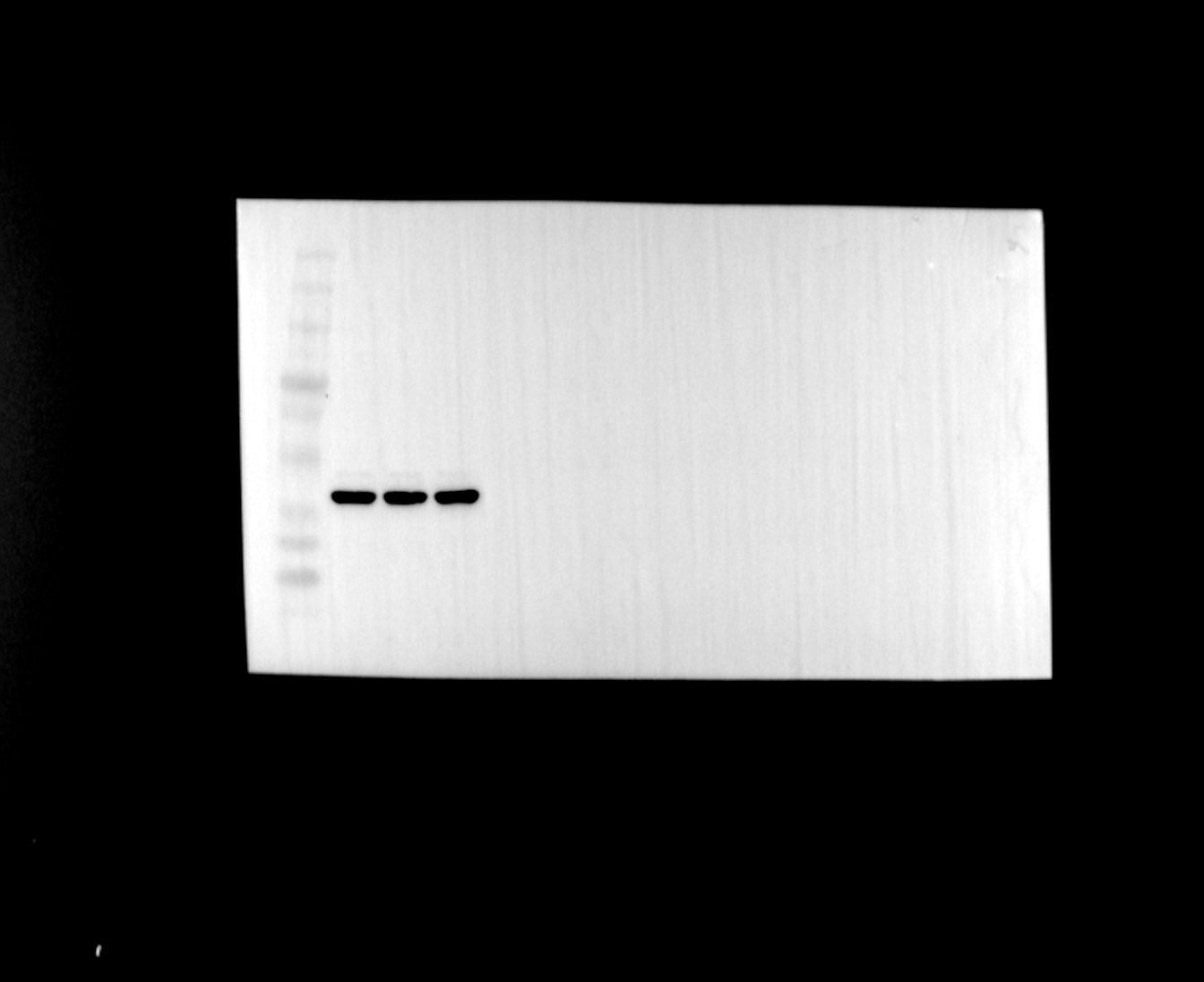

Supplement: Supplementary file 1 — Supplementary Material 1. [file 41065_2025_442_MOESM1_ESM.zip › Original image for western blot-marker/Original image Figure 4A/Figure 4A GAPDH.tif]

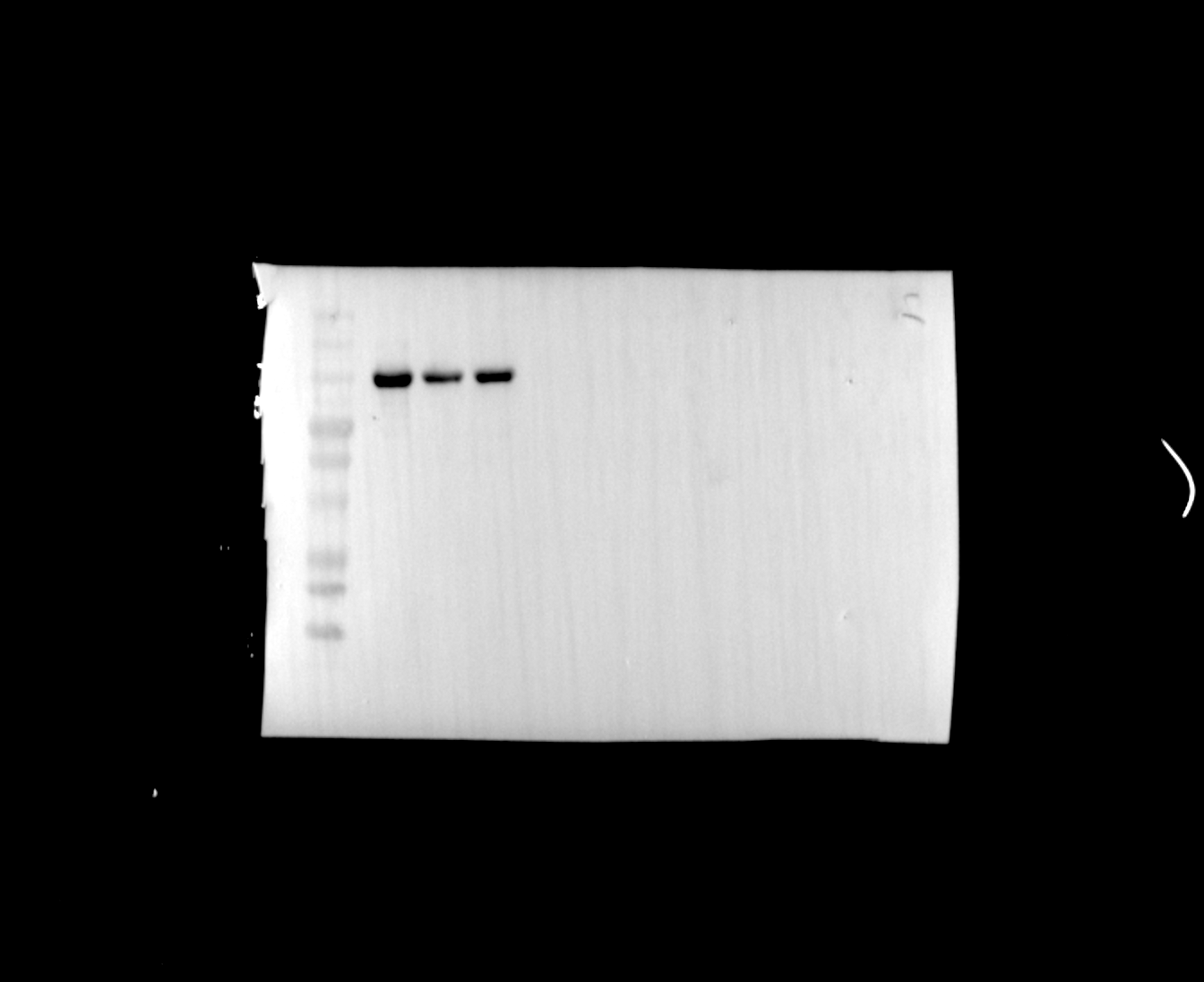

Supplement: Supplementary file 1 — Supplementary Material 1. [file 41065_2025_442_MOESM1_ESM.zip › Original image for western blot-marker/Original image Figure 4A/Figure 4A N-cadherin.tif]

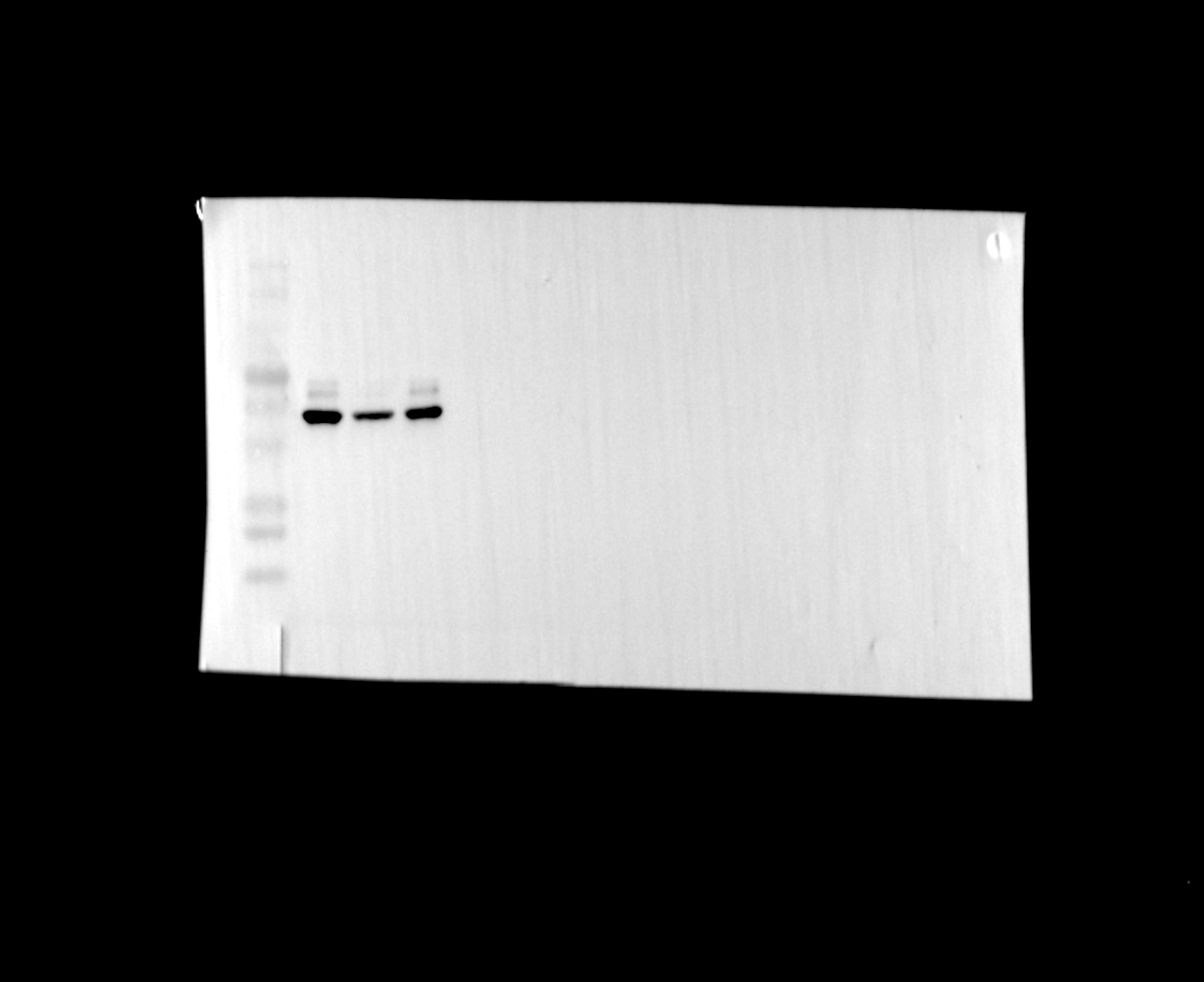

Supplement: Supplementary file 1 — Supplementary Material 1. [file 41065_2025_442_MOESM1_ESM.zip › Original image for western blot-marker/Original image Figure 4A/Figure 4A vimentin.tif]

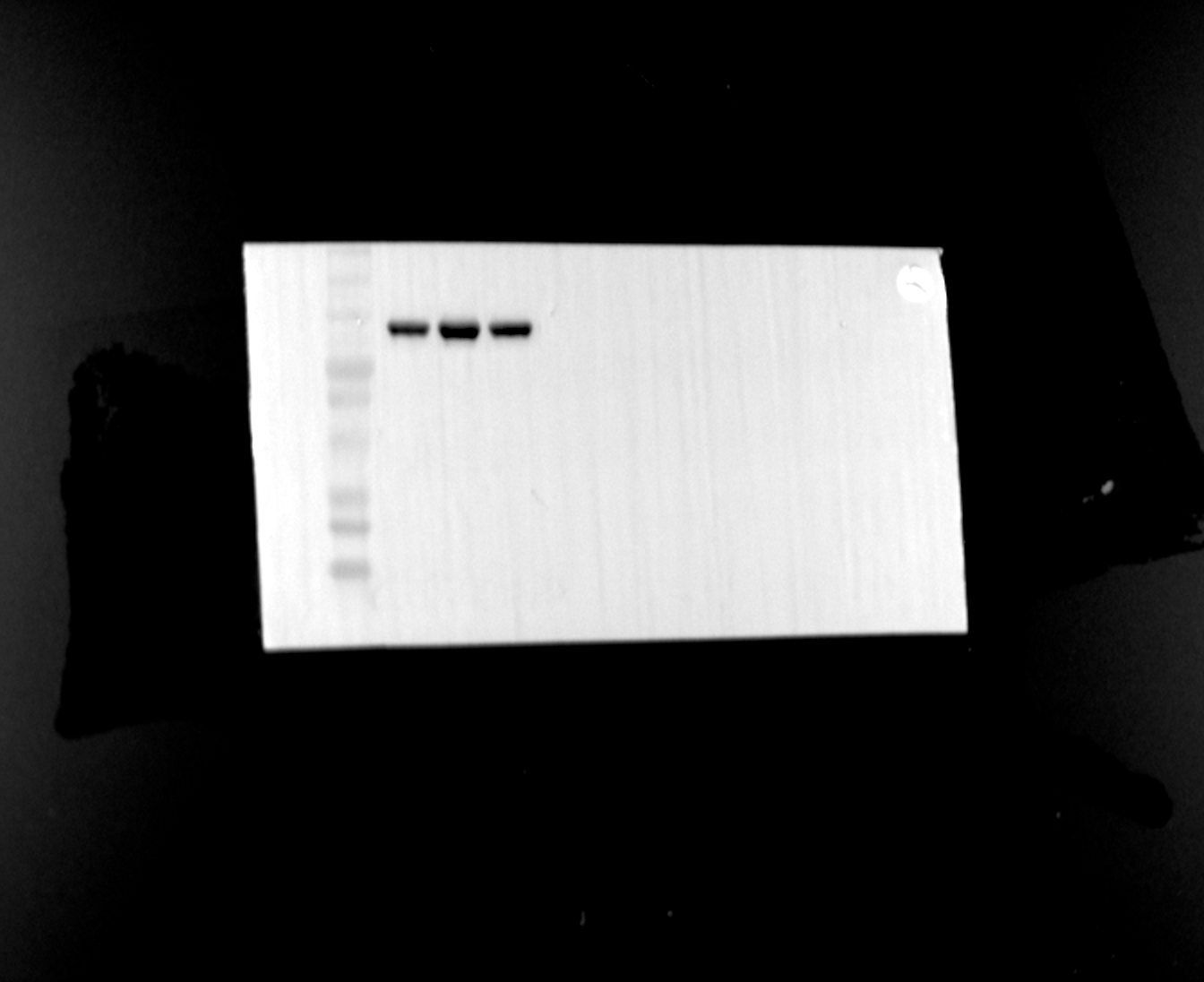

Supplement: Supplementary file 1 — Supplementary Material 1. [file 41065_2025_442_MOESM1_ESM.zip › Original image for western blot-marker/Original image Figure 4B/Figure 4B E-cadherin.tif]

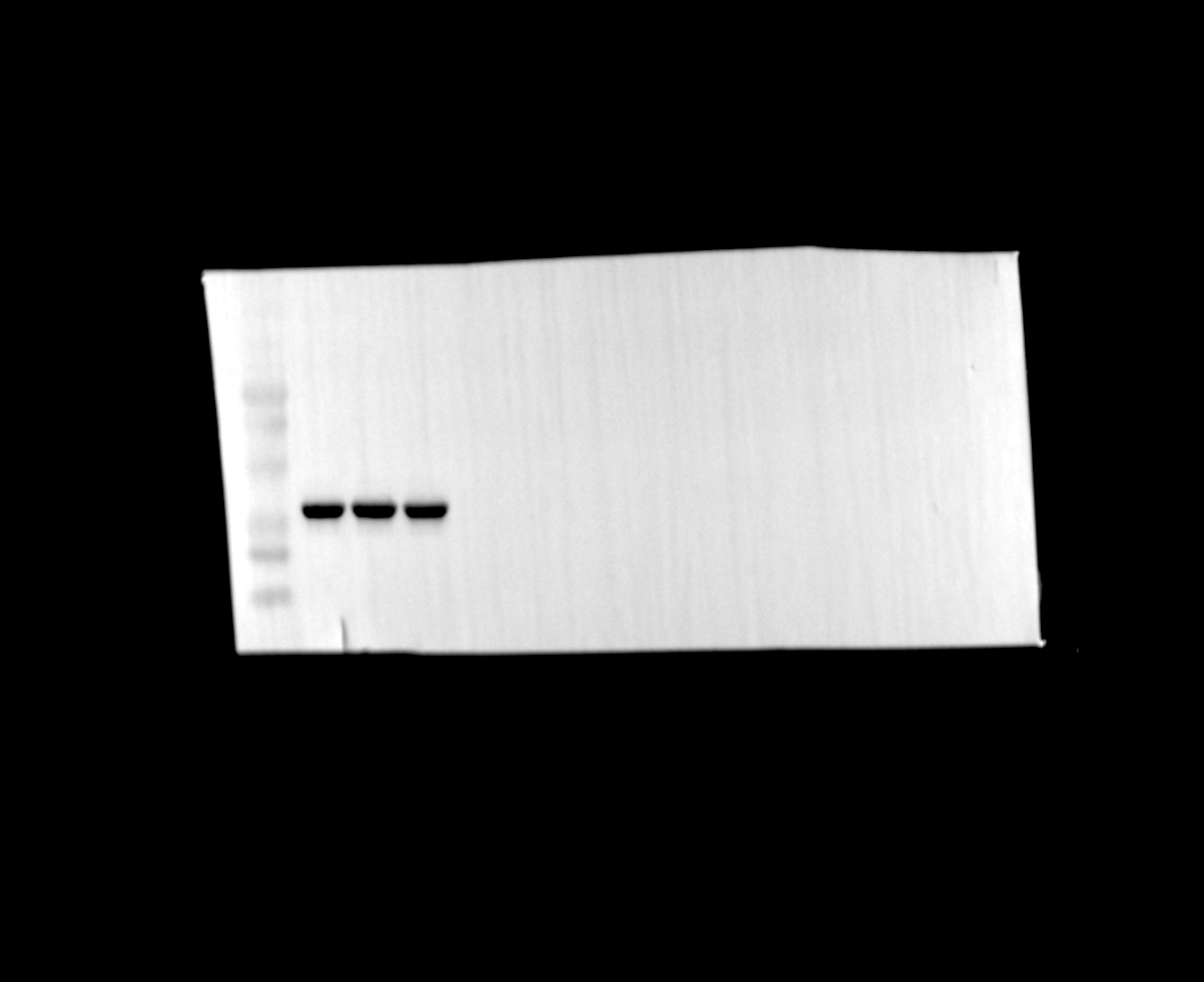

Supplement: Supplementary file 1 — Supplementary Material 1. [file 41065_2025_442_MOESM1_ESM.zip › Original image for western blot-marker/Original image Figure 4B/Figure 4B GAPDH.tif]

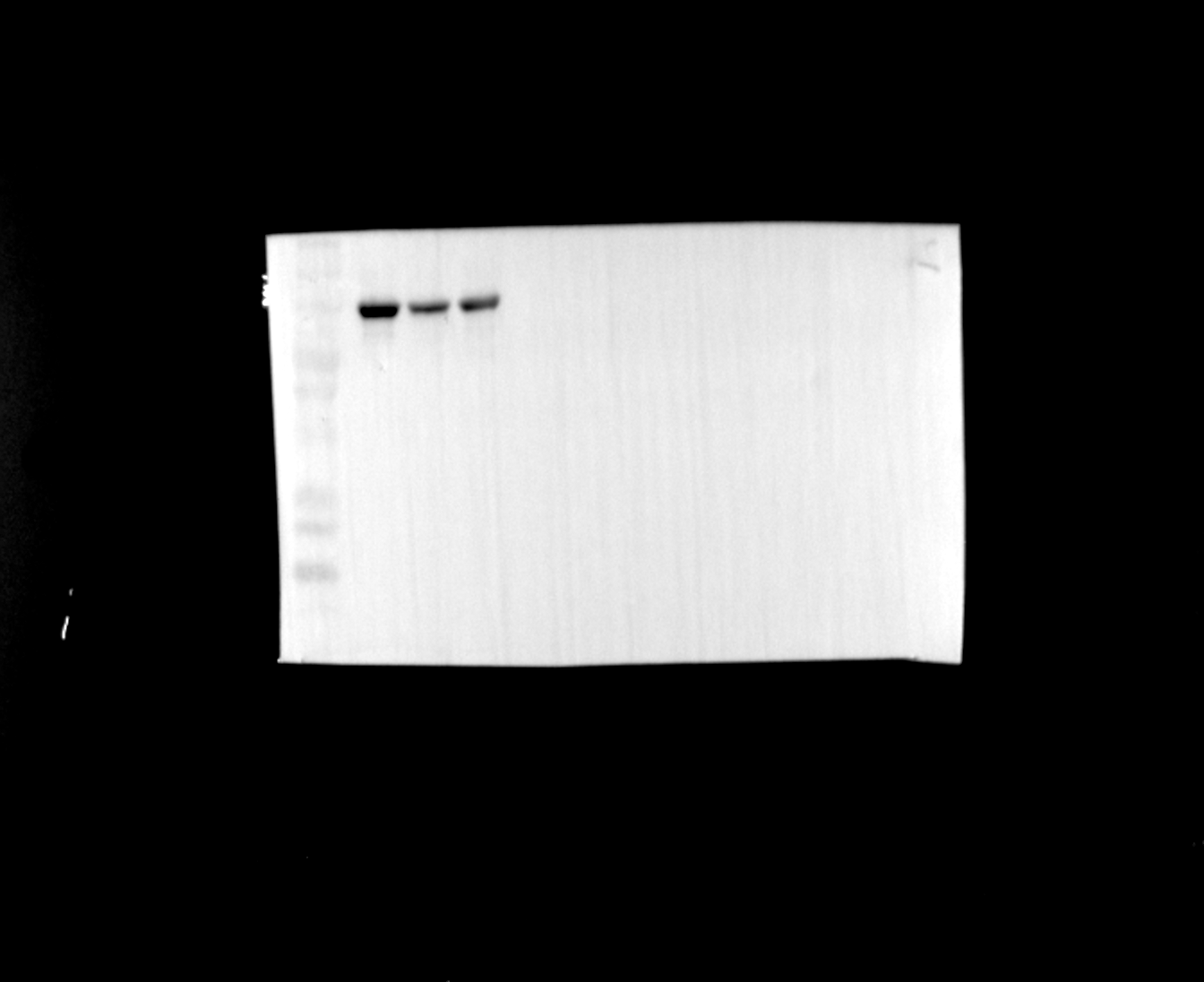

Supplement: Supplementary file 1 — Supplementary Material 1. [file 41065_2025_442_MOESM1_ESM.zip › Original image for western blot-marker/Original image Figure 4B/Figure 4B N-cadherin.tif]

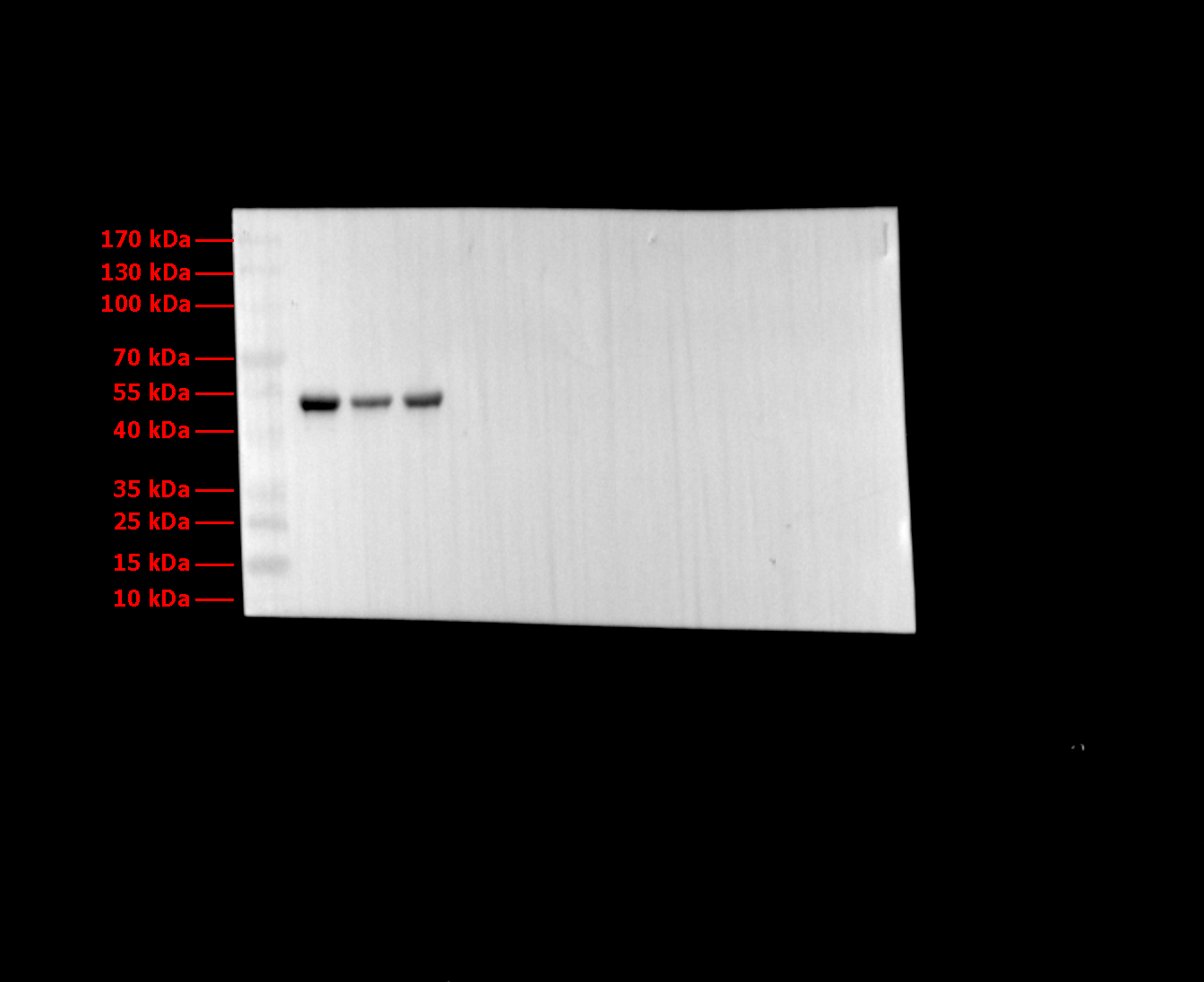

Supplement: Supplementary file 1 — Supplementary Material 1. [file 41065_2025_442_MOESM1_ESM.zip › Original image for western blot-marker/Original image Figure 4B/Figure 4B vimentin.tif]

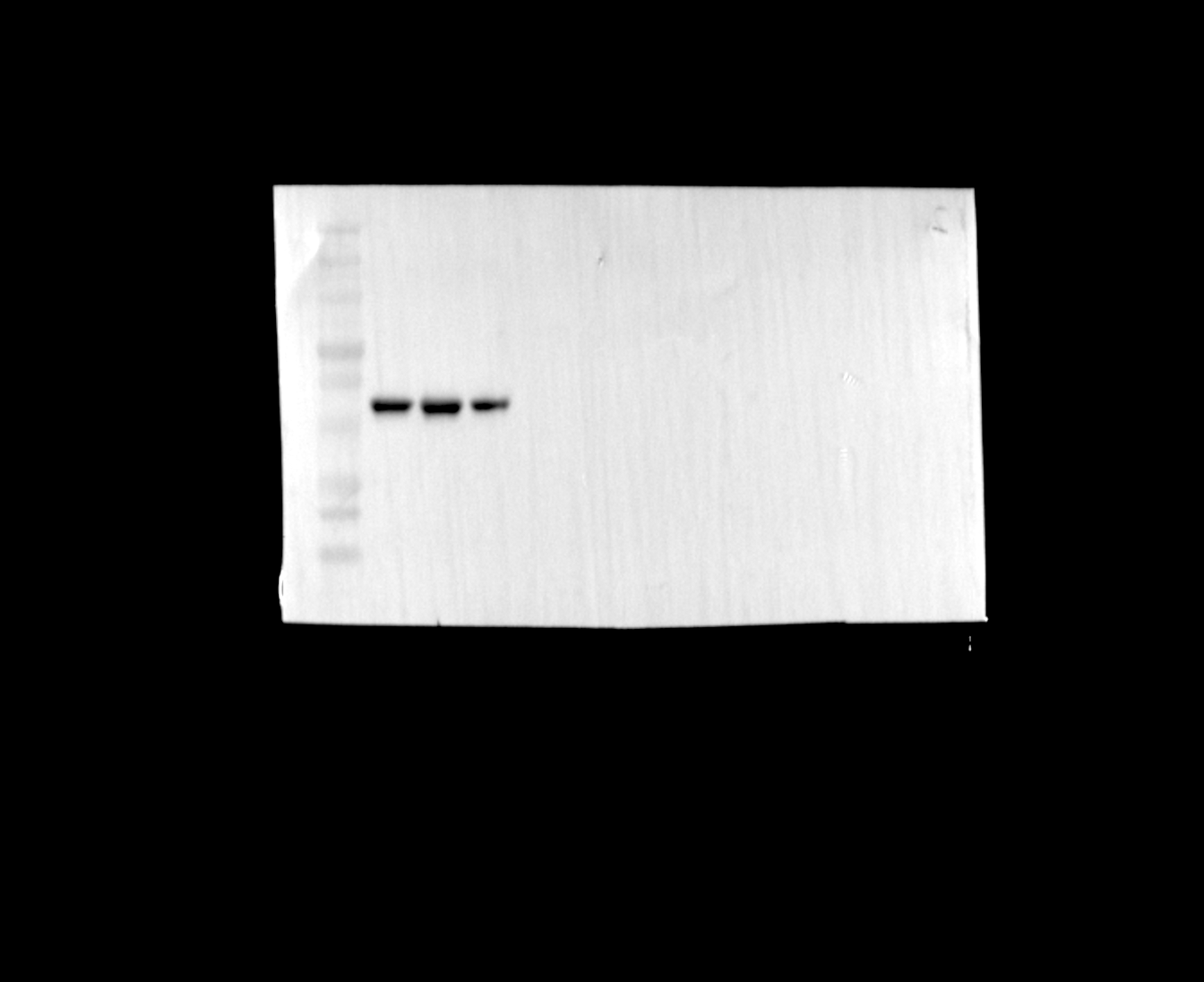

Supplement: Supplementary file 1 — Supplementary Material 1. [file 41065_2025_442_MOESM1_ESM.zip › Original image for western blot-marker/Original image Figure 5D/Figure 5D EIF4A3.tif]

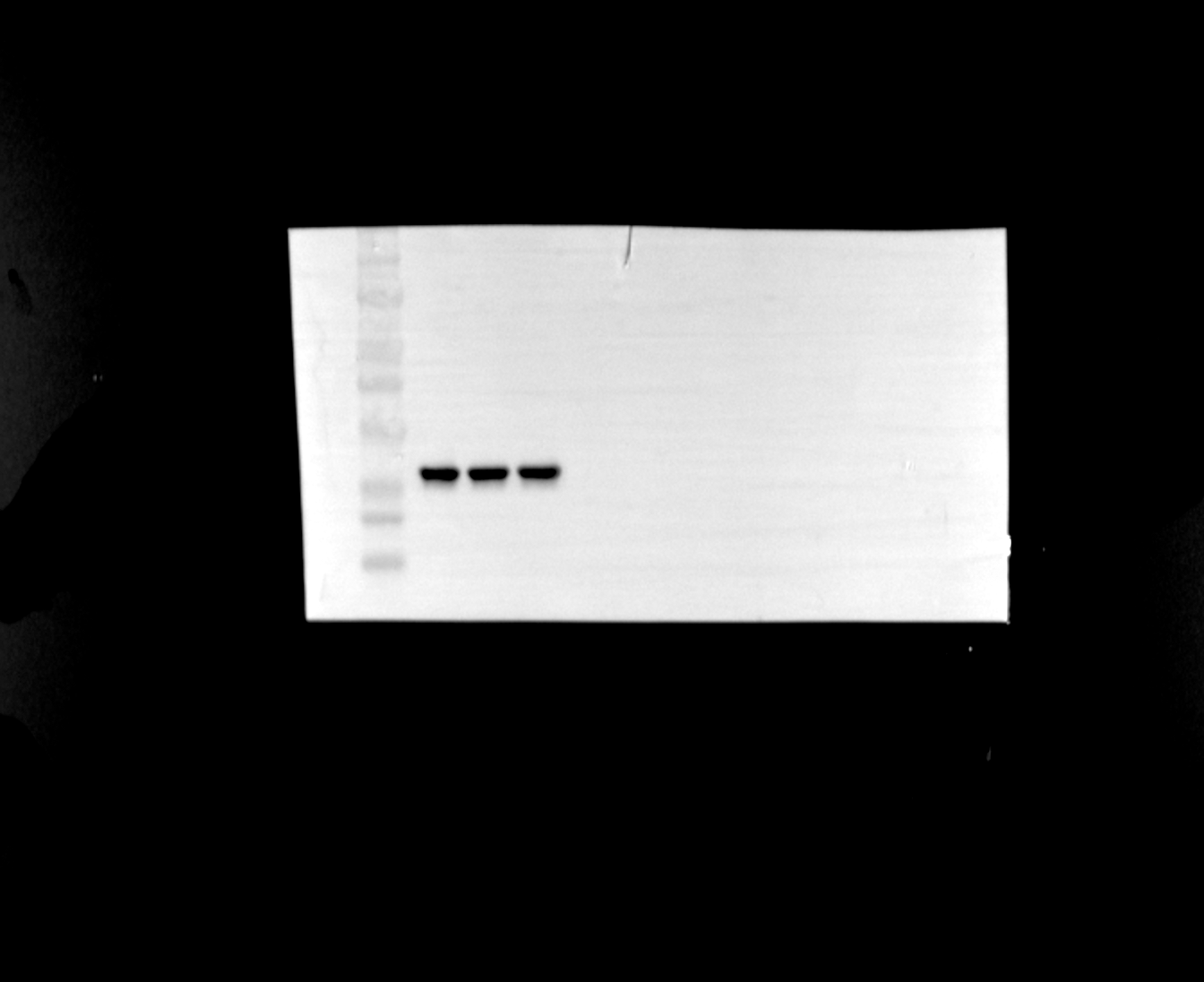

Supplement: Supplementary file 1 — Supplementary Material 1. [file 41065_2025_442_MOESM1_ESM.zip › Original image for western blot-marker/Original image Figure 5D/Figure 5D GAPDH.tif]

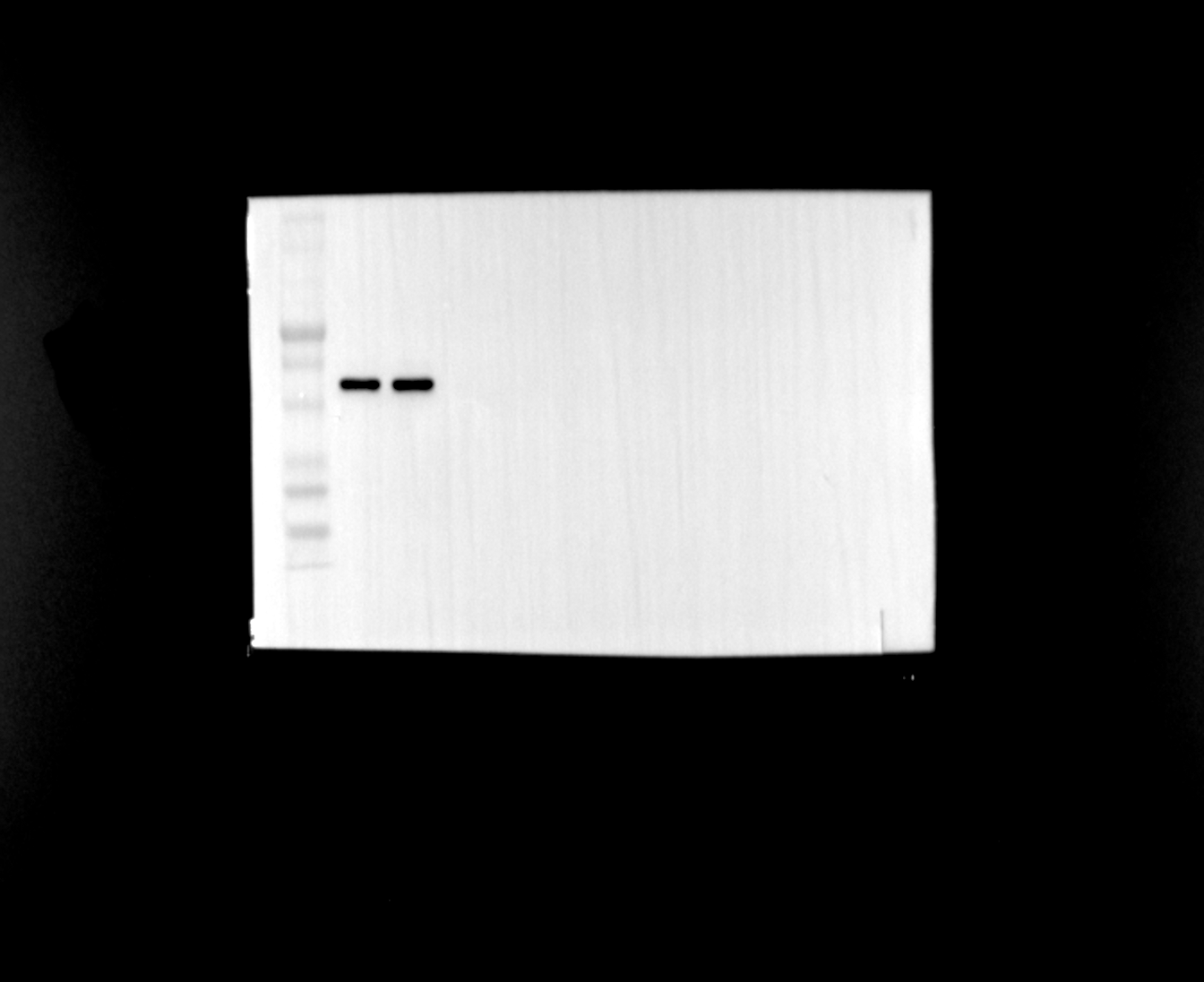

Supplement: Supplementary file 1 — Supplementary Material 1. [file 41065_2025_442_MOESM1_ESM.zip › Original image for western blot-marker/Original image Figure 6D/Figure 6D EIF4A3.tif]

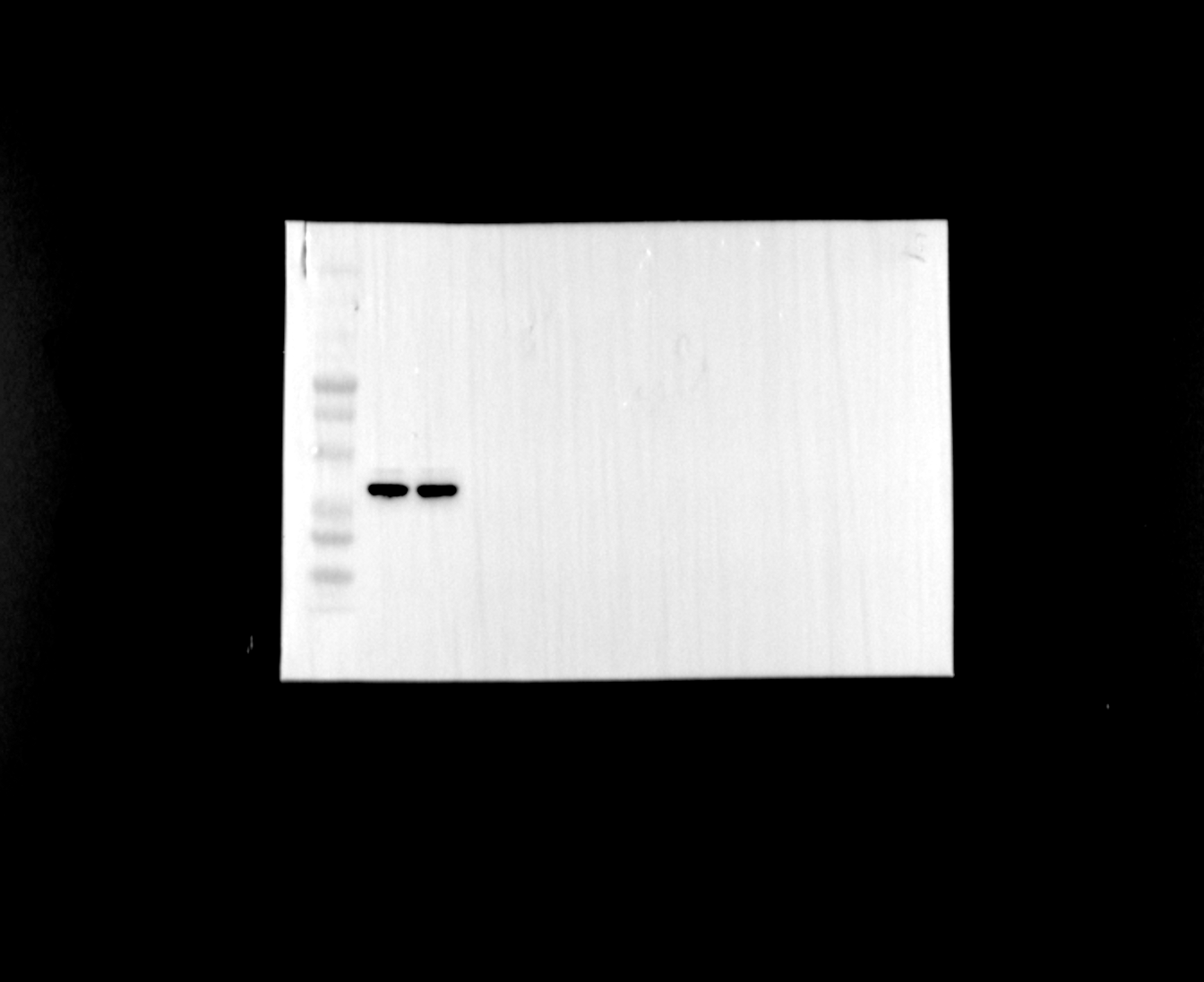

Supplement: Supplementary file 1 — Supplementary Material 1. [file 41065_2025_442_MOESM1_ESM.zip › Original image for western blot-marker/Original image Figure 6D/Figure 6D GAPDH.tif]

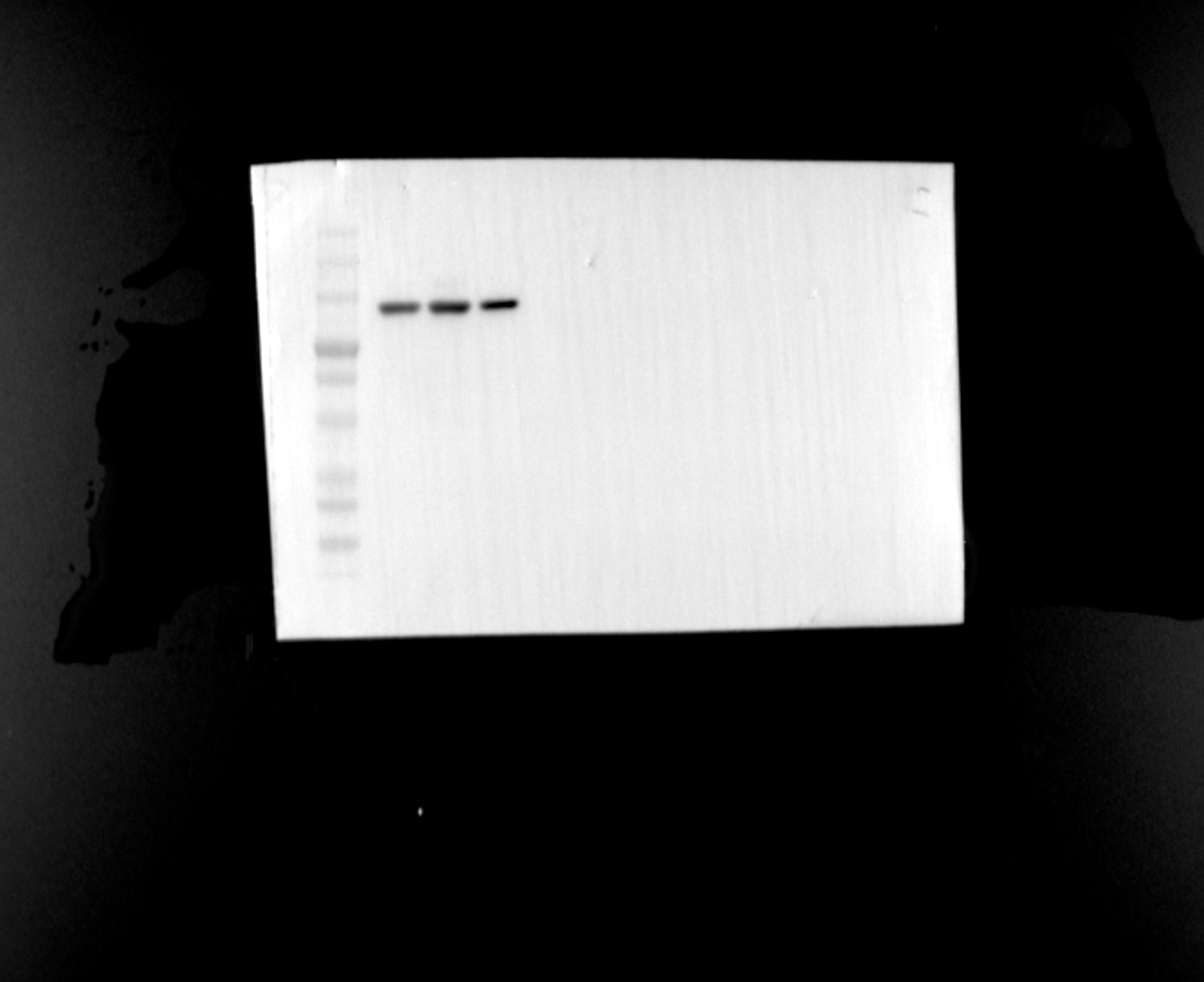

Supplement: Supplementary file 1 — Supplementary Material 1. [file 41065_2025_442_MOESM1_ESM.zip › Original image for western blot-marker/Original image Figure 6E/Figure 6E E-cadherin.tif]

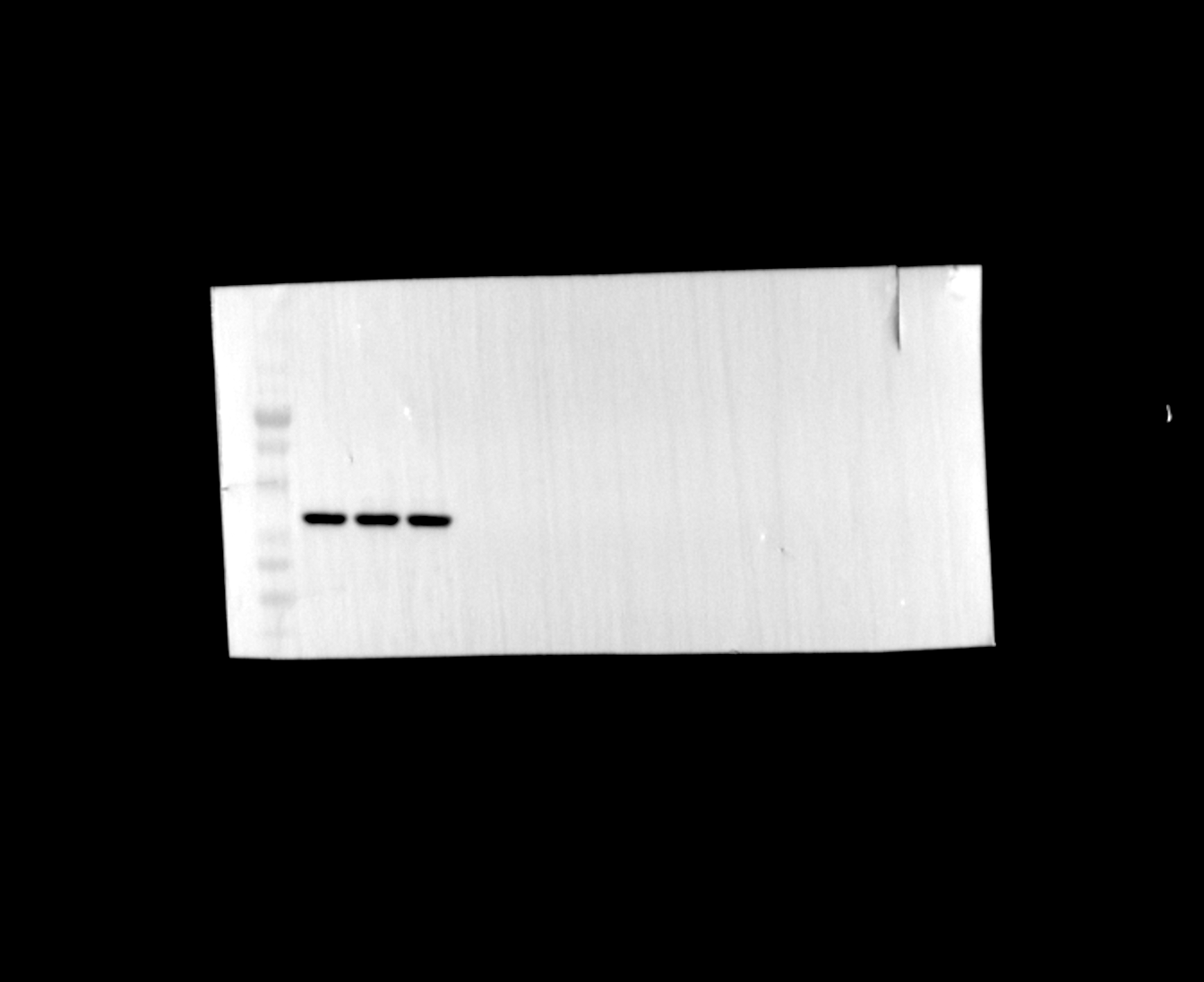

Supplement: Supplementary file 1 — Supplementary Material 1. [file 41065_2025_442_MOESM1_ESM.zip › Original image for western blot-marker/Original image Figure 6E/Figure 6E GAPDH.tif]

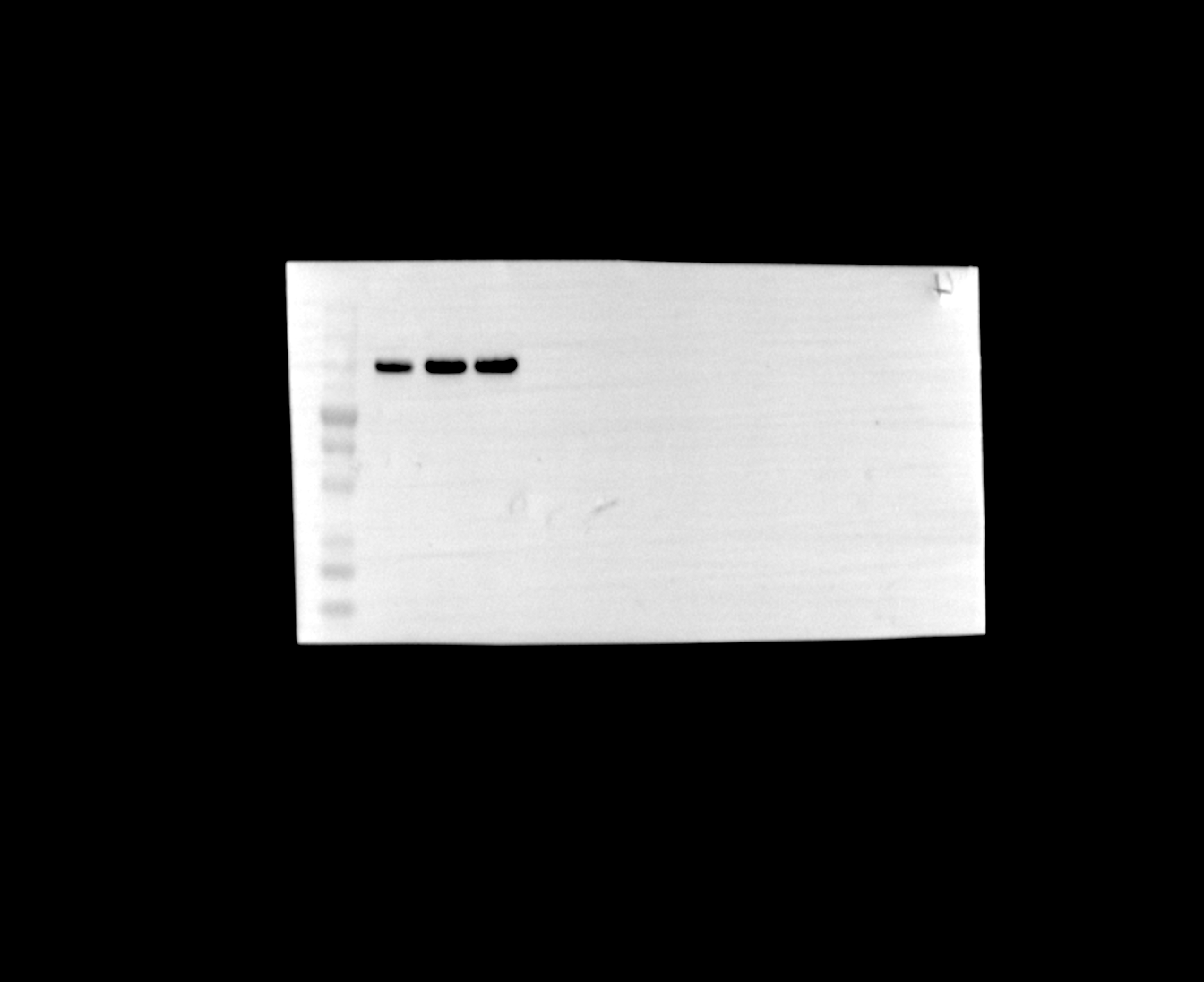

Supplement: Supplementary file 1 — Supplementary Material 1. [file 41065_2025_442_MOESM1_ESM.zip › Original image for western blot-marker/Original image Figure 6E/Figure 6E N-cadherin.tif]

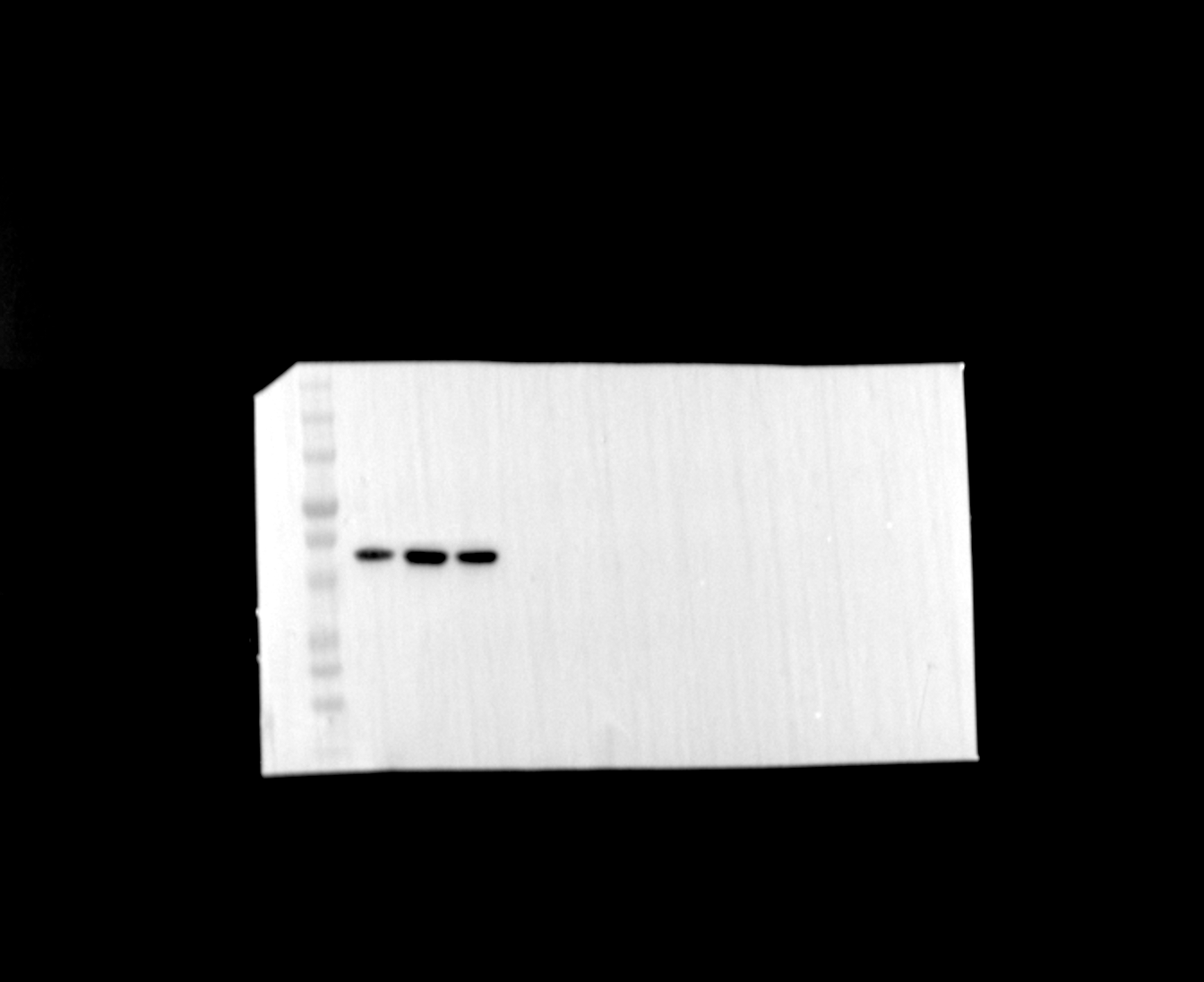

Supplement: Supplementary file 1 — Supplementary Material 1. [file 41065_2025_442_MOESM1_ESM.zip › Original image for western blot-marker/Original image Figure 6E/Figure 6E vimentin.tif]

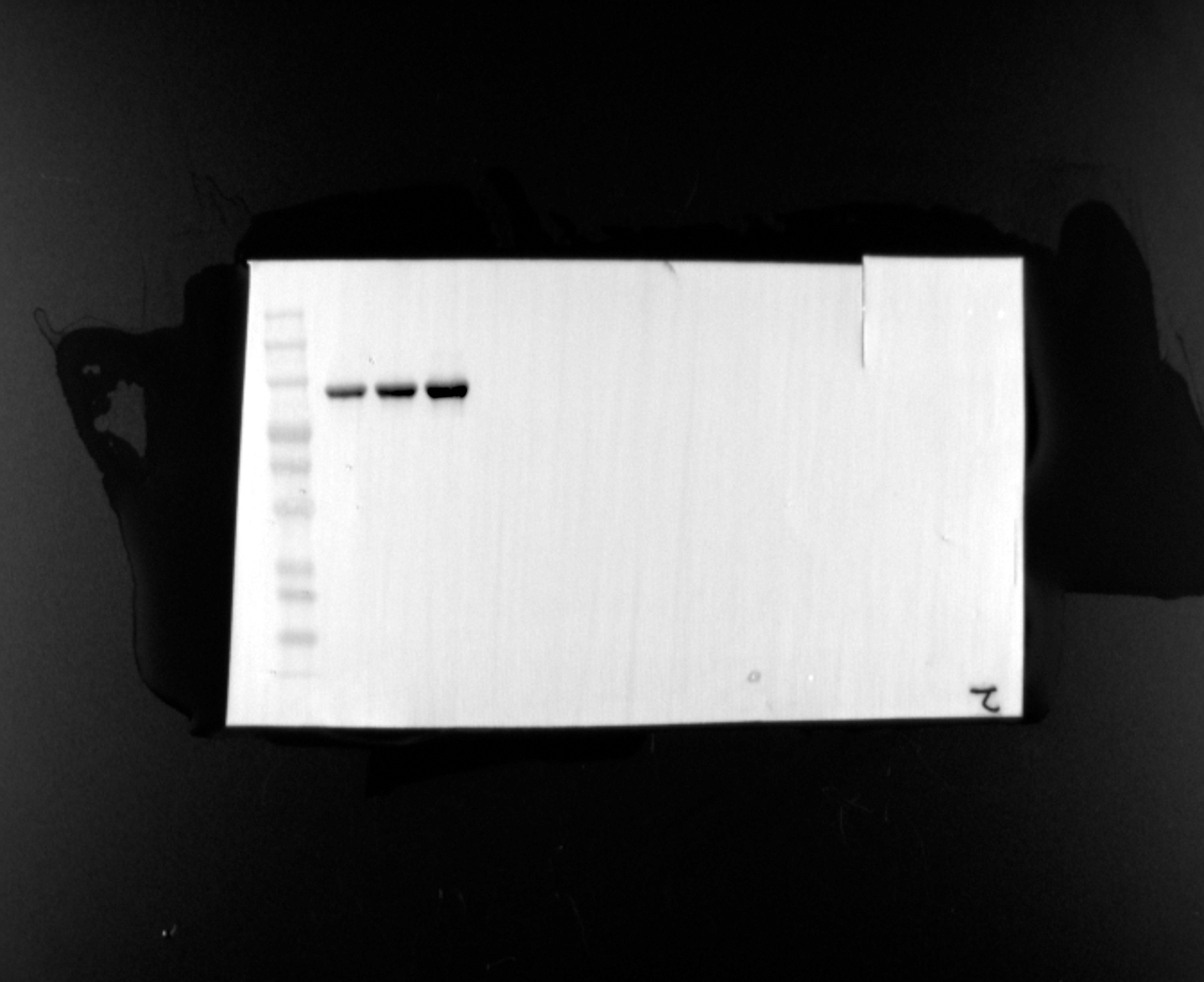

Supplement: Supplementary file 1 — Supplementary Material 1. [file 41065_2025_442_MOESM1_ESM.zip › Original image for western blot-marker/Original image Figure 6F/Figure 6F E-cadherin.tif]

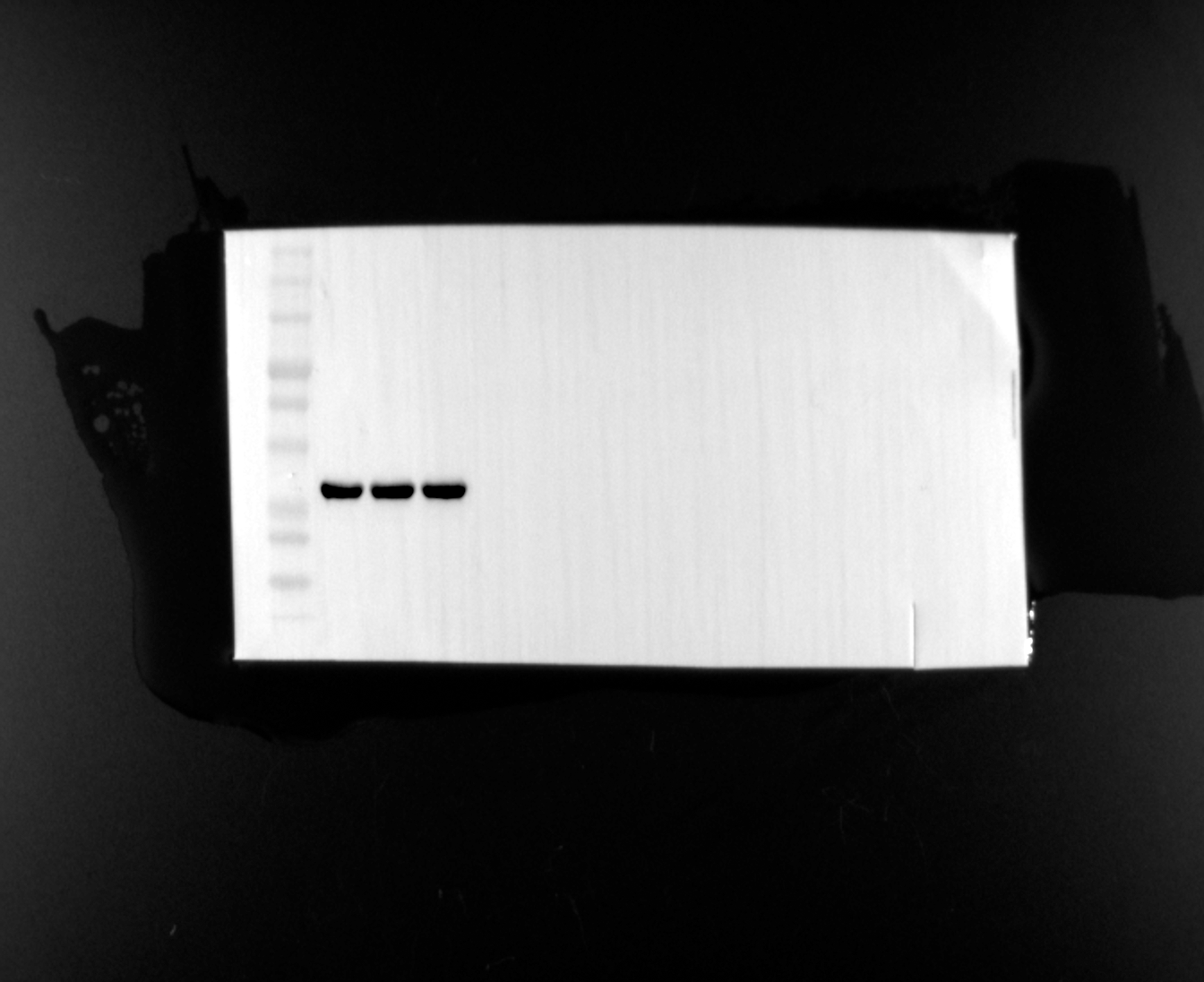

Supplement: Supplementary file 1 — Supplementary Material 1. [file 41065_2025_442_MOESM1_ESM.zip › Original image for western blot-marker/Original image Figure 6F/Figure 6F GAPDH.tif]

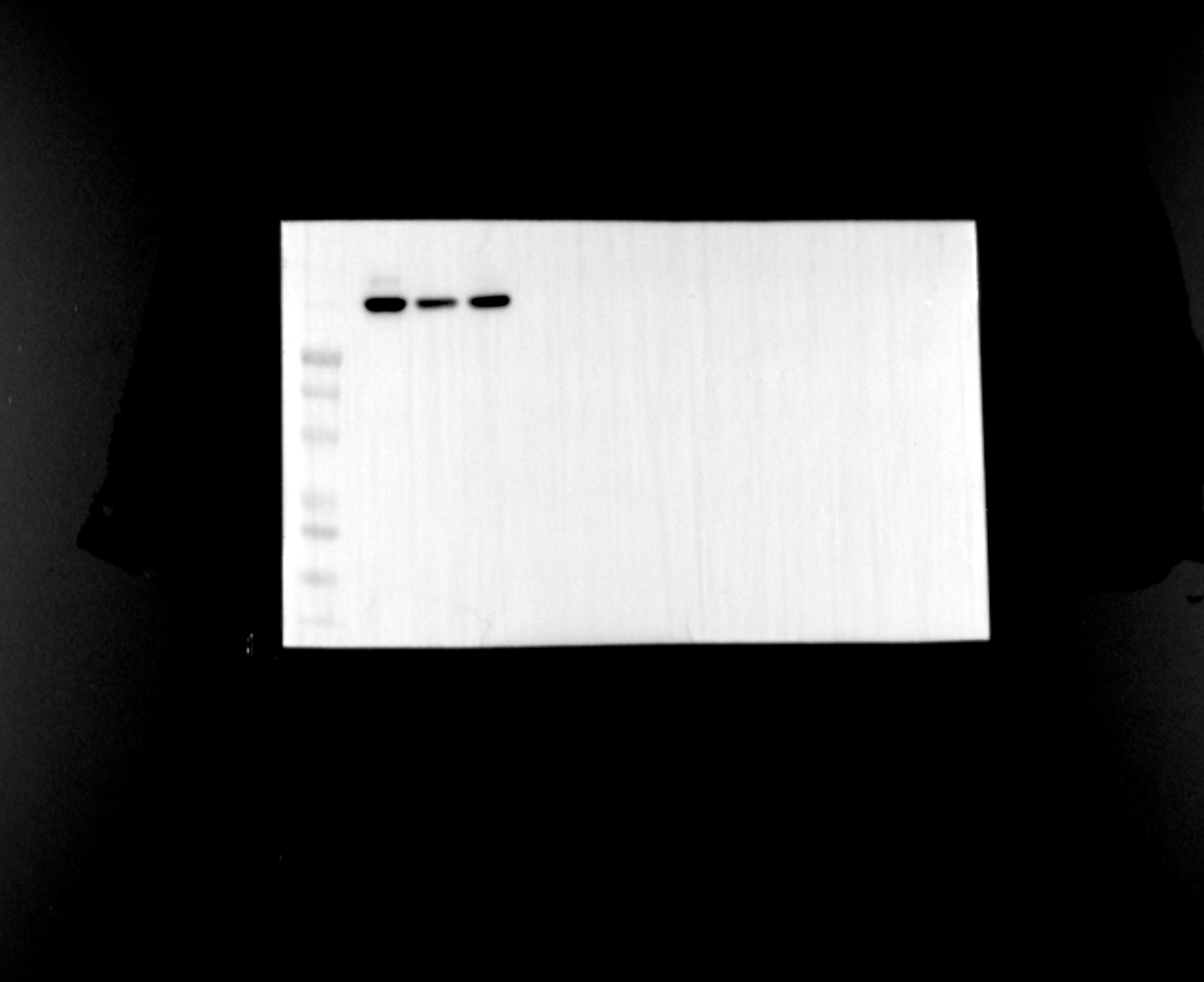

Supplement: Supplementary file 1 — Supplementary Material 1. [file 41065_2025_442_MOESM1_ESM.zip › Original image for western blot-marker/Original image Figure 6F/Figure 6F N-cadherin.tif]

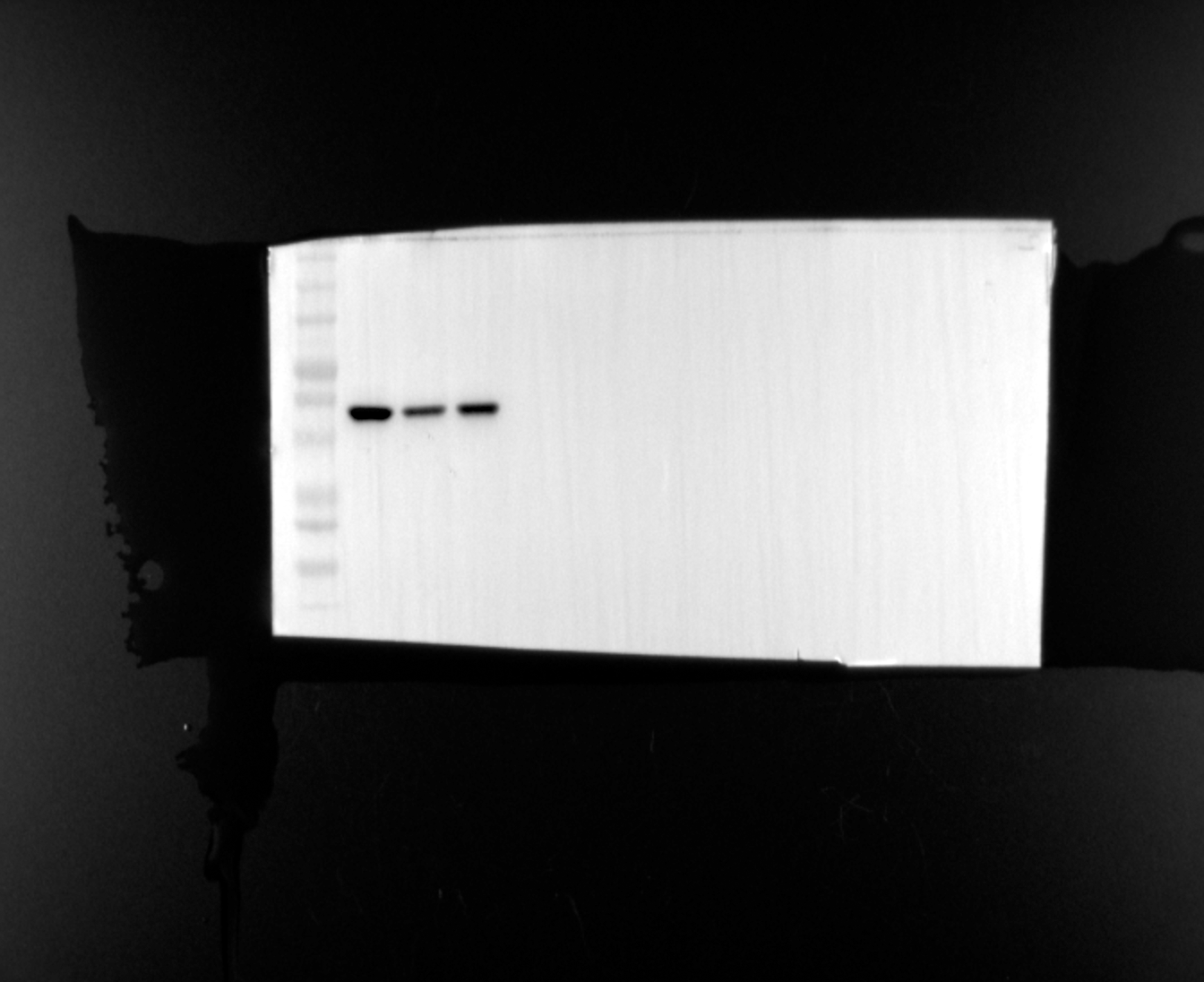

Supplement: Supplementary file 1 — Supplementary Material 1. [file 41065_2025_442_MOESM1_ESM.zip › Original image for western blot-marker/Original image Figure 6F/Figure 6F vimentin.tif]
